# The Articulation of Sauropod Necks: Methodology and Mythology

**DOI:** 10.1371/journal.pone.0078572

**Published:** 2013-10-30

**Authors:** Kent A. Stevens

**Affiliations:** Department of Computer and Information Science, University of Oregon, Eugene, Oregon, United States of America; Raymond M. Alf Museum of Paleontology, United States of America

## Abstract

Sauropods are often imagined to have held their heads high atop necks that ascended in a sweeping curve that was formed either intrinsically because of the shape of their vertebrae, or behaviorally by lifting the head, or both. Their necks are also popularly depicted in life with poses suggesting avian flexibility. The grounds for such interpretations are examined in terms of vertebral osteology, inferences about missing soft tissues, intervertebral flexibility, and behavior. Osteologically, the pronounced opisthocoely and conformal central and zygapophyseal articular surfaces strongly constrain the reconstruction of the cervical vertebral column. The sauropod cervico-dorsal vertebral column is essentially straight, in contrast to the curvature exhibited in those extant vertebrates that naturally hold their heads above rising necks. Regarding flexibility, extant vertebrates with homologous articular geometries preserve a degree of zygapophyseal overlap at the limits of deflection, a constraint that is further restricted by soft tissues. Sauropod necks, if similarly constrained, were capable of sweeping out large feeding surfaces, yet much less capable of retracting the head to explore the enclosed volume in an avian manner. Behaviorally, modern vertebrates generally assume characteristic neck postures which are close to the intrinsic curvature of the undeflected neck. With the exception of some vertebrates that can retract their heads to balance above their shoulders at rest (e.g., felids, lagomorphs, and some ratites), the undeflected neck generally predicts the default head height at rest and during locomotion.

## Introduction

Sauropod necks were but one remarkable aspect of an altogether remarkable vertebrate. The necks of many sauropod taxa comprised a larger proportion of the presacral axial skeleton than found in any extant non-avian, with individual vertebrae representing extremes of pneumaticity, elongation, and size. The combination of relatively tiny head, elongate neck, and enormous body has posed fascinating questions regarding sauropods, including how they fed, moved, and simply how they appeared in life.

Since only their fossilized bones remain, and usually incomplete and imperfectly preserved at that, even their skeletal reconstructions have been subject to differences in interpretation and sometimes artistic liberties. The overall bauplan of these great giants remains controversial (see below), let alone how they might have held their heads and used their necks in life. Settling the essential questions of sauropod feeding habits and the role of their remarkable necks in feeding will be challenging, since the origin and role of the long neck of the giraffe remains controversial despite their being available for direct observation, as living and behaving animals. Even if alive today, some contention could be expected regarding how the sauropod got its long, long neck. But given only their fossils, much must be inferred and little can be observed directly. This review attempts to summarize what can be concluded about sauropod neck articulation based on correlations between function and (osteological) form. The methodology is necessarily inferential and incremental, accumulating a coherent explanation that, while highly incomplete and speculative, is at least consonant with what can be derived from other sources.

### What if Giraffes Were Extinct?

**Figure 1:**
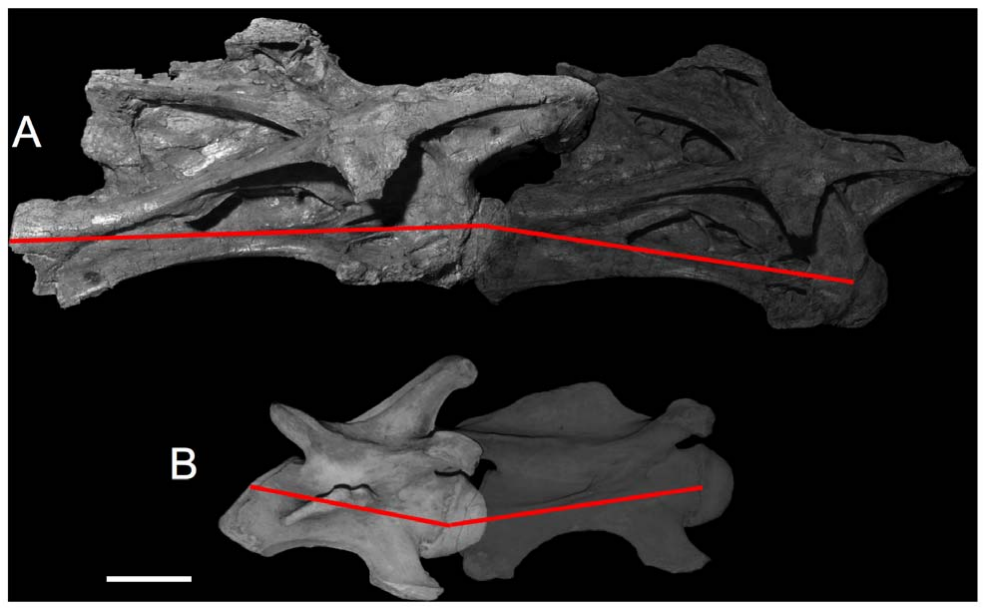
Intrinsic neck curvature starts with the bones. In (A), cervical vertebrae C4 and C5 of Giraffatitan brancai specimen SI are shown articulated and undeflected, i.e., in osteologically neutral pose (ONP). Their vertebral axes, shown in red, naturally create a slight downward bend in ONP, contributing to the subtle ventral osteologically induced curvature (OIC) likely shared with other sauropod necks cranially (Figure 5). In (B) the giraffe Giraffa camelopardalis, cervical vertebrae C6 and C7 are shown also in ONP, revealing the naturally-ascending slope characteristic of giraffe necks at the base. Note the similarity in their opisthocoelous central articulations compared to the sauropod above. Vertebrae to scale; scale bar equals 10 cm. Giraffatitan photographs courtesy Christopher McGowan; giraffe photographs courtesy Brian Curtice.

By analogy, imagine that giraffes were extinct, and known only by their desiccated bones, with some cervical columns remaining in apparently close articulation but in opisthotonic pose [1], while others are found associated but disarticulated. The skeletal structure could still be reconstructed with reasonable accuracy to reveal the overall conformation of the axial skeleton, however, revealing the abrupt rise of the neck at the shoulders, the straight mid-neck, the downward-tilted skull, and the resultant head height – all surmised by reassembling the bones with the joints in their neural, undeflected state (Figure 1). The neck's range of motion could then be explored by re-articulating the cervical vertebrae (spaced appropriately to account for the missing cartilage), revealing differences in lateral versus dorsoventral flexibility, and variation in flexibility along the length of the neck (the consequences of which are observable in life). Suspicion would likely arise that the neck's limited ventral flexibility posed a problem for reaching down to water, requiring splayed forelimbs or bent elbows. As will be reviewed, preventing disarticulation ultimately limits joint range of motion, and soft tissues further constrains flexibility. Ligamentous synovial capsules surrounding the zygapophyses arrest deflection prior to their disarticulation, plus layers of deep and superficial musculature and fascia would further restrict the effective range of motion (see below). While joint geometry may allow some estimation of joint flexibility and provide some insight into posture and feeding envelope, it would not go far towards revealing behavioral specializations in feeding or other roles of the neck. This is the situation faced by paleontologists when seeking answers to just those questions for sauropods. While estimating sauropod neck curvature and flexibility from fossilized bones based on extant models is necessarily speculative, support is unfortunately even more tenuous regarding speculations related to behavior.

### The Necessity of Speculation

Unlike other sciences where the subjects under study are extant and their behaviors (e.g., physical, chemical, psychological, societal) can be observed directly, in paleontology, when the subjects are extinct their behaviors can only be inferred. The process of scientific inference regarding extinct behavior is therefore indirect and conjectural, and many of the presumptions about how these animals lived out their lives rely on tacit intuition and the selection of modern examples that appear to support a given conjecture.

Since their first discovery, sauropods have long been the subject of speculation, e.g., that they had a sprawling stance [2], [3], used their long necks as snorkels while walking along the bottoms of lakes [4], walked bipedally [5], [6]), used their tails as supersonic bullwhips [7], used their hindlimbs to kick predators [8], sat down to eat [9], held their heads so high as to require multiple hearts to create sufficient blood pressure [10], [11], and long necked or short, habitually dorsiflexed the base of their long necks to achieve maximum head elevation [12].

Note that the above speculations are suggestions not only about potential function (snorkeling, standing on hind legs, whipping, kicking, sitting, and holding the head high) but about behavior, and unless outright refuted as physically impossible, are subsequently adopted – or not – based on their appeal and support by analogical reasoning (see below). There is also a tendency to propose conjectures that are not scientifically testable (i.e., not refutable) yet seem compelling, popular, and make for a satisfactory story [13]. For example, Paul [14] argues that “… a low neck increases the risk of not spotting attackers … and so appears illogical”. Long necks that reach high allow the owner to see approaching predators, to see where they are going, to eat what others with shorter necks could not reach, and to keep their necks out of reach of predators' jaws [15]. For sauropods to have not used their long necks to elevate the head, and to keep it elevated habitually when not drinking or browsing low vegetation, would have been to miss the best part of having a long neck. Even sauropods such as Diplodocus with shorter forelimbs (and seemingly ill-adapted to a life of high browsing) are expected to have raised their heads skyward habitually (by bending the neck sharply upward at the base and tucking the chin down to level out at the head) [12]. While sauropods with soaring necks is congruous with childhood expectations, these often-repeated and seldom-challenged speculations amount to little more than scientific mythology.

Recently, however, there has been increasing use of a method to challenge the mythology using observations of modern vertebrates and certain bridging assumptions to ‘ground-truth’ proposals about sauropod pose, flexibility, and behavior. The application of this methodology to examine the mythology is the subject of this review.

Conjectures about sauropod neck function, physiology, and feeding behavior are invariably based on skeletal reconstructions by illustrations or mounts and while such reconstructions are very familiar and seemingly authoritative, they often amount to hypotheses or conjectures incorporating significant artistic interpretation (see below). Some effort will be devoted to this issue, since sauropod reconstructions, whether physical or pictorial, are often used uncritically, as will be discussed. The relationships between osteological form and biological function in general, and of vertebral articular geometry and joint articulation in particular are becoming increasingly understood, and recent studies are confirming that vertebral osteology can tell us something about pose and flexibility. The vertebrate neck is not merely a chain of bones and joints, but a system, and observable correlates between structure and function in the necks of extant vertebrates are becoming better understood, thus permitting more principled application towards interpreting sauropod neck function.

### The Appeal of Simple Explanations

“… the truth will out. Nature's phenomena will agree or they'll disagree with your theory. Although you may gain some temporary fame and excitement, you will not gain a good reputation as a scientist if you haven't tried to be very careful…” [16].

There seems a universal tendency to confer greater trust upon simple parsimonious explanations of natural phenomena, those that capture some essence in few words and to hold broadly without exception. It has been suggested that, based on how extant amniotes hold their heads in alert rest, that sauropods assumed a posture with the neck is maximally extended (dorsiflexed) at the base and the head maximally ventriflexed [12], [17], giving weight to the popular expectation that sauropods indeed held their heads up. Disregarding for the moment whether amniotes actually raise their heads maximally when in alert rest, it represents an attempt to ground speculations by more than just an appeal to common sense. Whether sauropods held their heads in such a state is not expected to be directly testable i.e., refutable. Instead, an indirect argument is provided based on an observable (and refutable) relationship in extant organisms to support the untestable speculation. In brief, the method, the ‘Extent Phylogenetic Bracket’, or EPB (discussed in more detail below). In brief, if a given unpreserved property such as a behavior is exhibited by an specifically-defined extant cohort of related organisms, there is reason to speculate that the extinct organism also shared this property. But as cautioned by the proponents of this approach [18], [19], even speculations that are successfully supported by the method remain guesses, only more ‘educated’ or ‘informed’ guesses. Following Witmer [19] “… the term speculation is not used here in its more common, pejorative sense, and implies no de facto absence of testability … we need greater methodological rigor in order to determine the limits of our objective inferences – that is, to constrain, not completely eliminate, speculation.” The EPB method provides at least some grounds for speculations that, by their very nature, cannot be empirically verified.

### Polarized Conjectures

The necessarily speculative nature of theorizing in paleontology is unfortunately susceptible to the social phenomenon of ‘group polarization’, as speculations are adopted and repeated by secondary sources [21], [22], [23]: “… as individuals learn that most of the other group members lean in one direction on some issue, they may adopt a more extreme attitude in the same direction” [24]. Group polarization may create a false dichotomy when outgroup opinions are stereotyped and misrepresented in stating the strengths of one idea or the weaknesses of another, and a general failure to acknowledge implicit bridging assumptions, exceptions, and potential pitfalls in subsequent citations.

**Figure 2:**
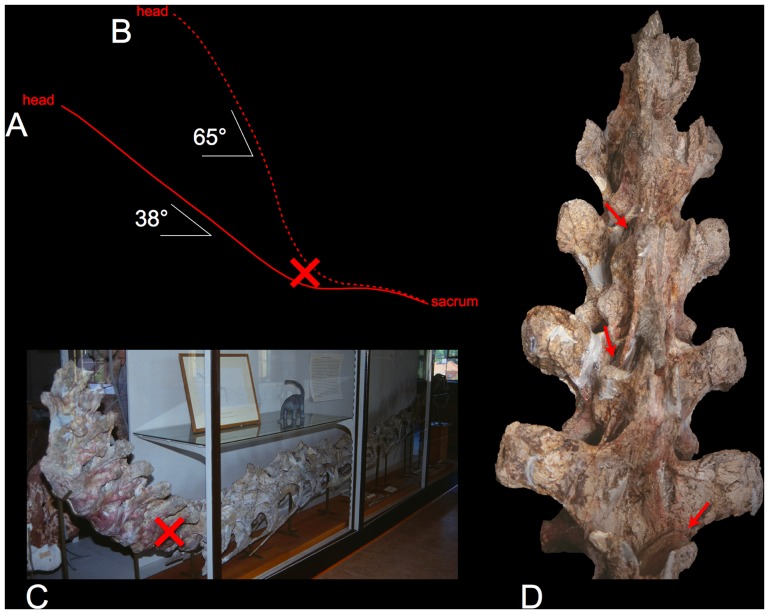
The life and death of *Euhelopus zdanskyi*. In 1929, Wiman illustrated this sauropod in life with a decidedly giraffe-like pose, rising at a slope of 38° (vertebral axes indicated by the solid red line in A, derived from [4:fig. 3 and pl. 3], see also [32:fig. 9]). In the life reconstruction the base of the neck was given the same curvature as the opisthotonic pose in which the original specimen was found (C). It has subsequently been depicted with a steeper slope (dashed red line in B, from [14]) that even exceeds the death pose in which bone already contacts bone (indicated by the red arrows in D). While the neck has also been regarded as more moderately curved [33], Euhelopus may in fact have had a straight neck in the cervico-dorsal region in ONP [31]–[32]. Photographs courtesy Valérie Marin-Rolland of the E. zdanskyi specimen PMU 24705, Paleontological Museum of Uppsala University.

**Figure 3:**
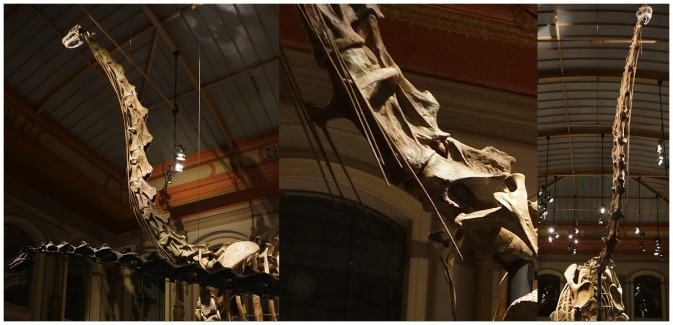
Impressive sculpture. The Giraffatitan brancai mount at the Humboldt Museum of Natural History has been restored with an extraordinarily steep neck at the base, with an ascending neck that appears to be in ONP. While the neural arches in the cervico-dorsal region were not preserved, the centra were, and the sculpture in the mounted skeleton deviates significantly from the actual fossil material (see Figure 4). Photographs by the author.

**Figure 4:**
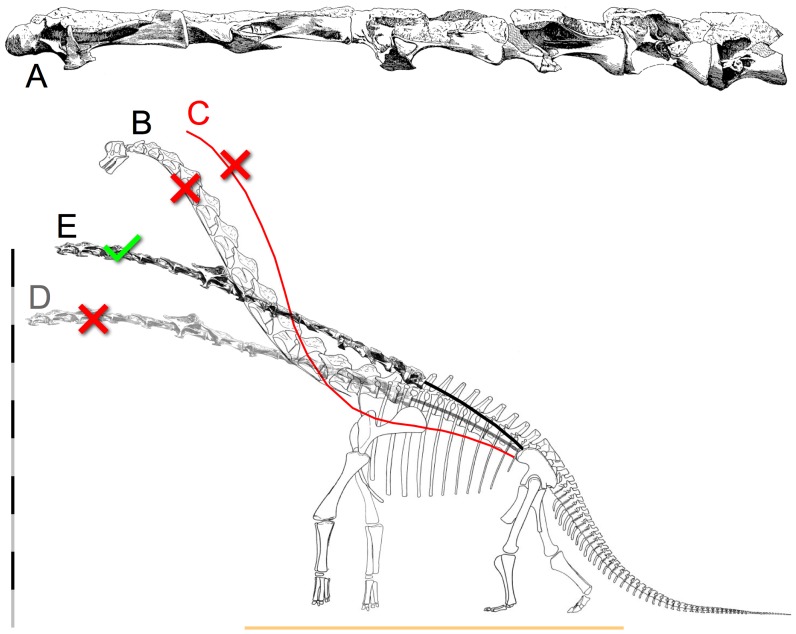
The iconic swan neck of *Giraffatitan brancai*. Janensch [104] (A) illustrated the original fossil material in the cervico-dorsal vertebrae (C10 to D2) as they were found, in articulation, and despite their missing the neural spines, the centra are collinear and appear close to ONP based on their central articulations. Janensch's skeletal reconstruction [27] (B), however, does not reflect this osteology; instead a gracefully-curved swan neck was illustrated, complete with restoring the vertebrae at the base of the neck as if wedge-shaped to formed that elegant rising curve in ONP. The slope of the neck increased further in some later illustrations, e.g., the red curve (C) is drawn from Paul's reconstruction [14; 32:fig. 6]. The centra at the base of the neck are straight, elongated cylinders with parallel anterior and posterior central margins (A) and not wedge-shaped with convergent margins (as inevitably, mis-represented) like those of a giraffe, there is no osteologically-induced bend at the base of the neck. Substituting an ONP reconstruction of the complete vertebral series from C3 to D2 based entirely on Janensch's individual vertebral illustrations (see text) two alternatives are presented (D and E). In D the slope of the anterior column matches that of the original skeletal reconstruction by Janensch [27], which has relatively high placement of the pectoral girdles upon the ribcage (but lower placement than Paul [14] illustrated, which caused his reconstruction to have a lower vertebral column at the base of the neck). If the scapulocoracoids are reconstructed as closely separated medially and more ventrally placed upon the ribcage, the resultant slope of the anterior dorsals rises necessarily. This raises the head height to 10 m, while the Berlin mount goes to 11, or more. Scale bar is 10 m. The horizontal line represents the ground plane according to revised appendicular reconstructions.

Regarding sauropod necks, for instance, while historically they have long been depicted with a wide range of combinations neck curvature (from straight to sharply reflex-curved) and slope at mid-neck (from horizontal, or even downward-sloping to vertical or even past vertical) [25], [26], [4], [14], [27], [28], [29], [30]–[33], there is a tendency for subsequent retrospectives and reviews to categorize, to simplify, and to polarize: “Sauropods can be broadly grouped into forms with … a presumably upright neck … and forms … with a presumably more horizontal neck” [34]. The expectation for increasingly high head elevation is exemplified by Euhelopus zdanskyi (Figure 2) which was originally depicted in 1929 with a slope of 38° [4] but that was later revised to 68° [14]. Mamenchisaurus hochuanensis was originally depicted with a descending neck [35], yet mamenchisaurids have subsequently been illustrated [14], [29] and mounted [Zigong Dinosaur Museum] with subvertical necks. Even Opisthocoelicaudia sharzynskii, found without a neck and originally reconstructed with a horizontal neck [36] was later given a swan-like neck [14], [29]. Giraffatitan brancai, perhaps the iconic swan-necked sauropod, was given one when first described and mounted has been been depicted with increasingly steep neck in both mounts (Figure 3) and illustrations (Figure 4) [27], [14], [29]. Camarasaurus was originally depicted without a swan neck [37], but soon acquired one [38], and to this day, camarasaurids are generally reconstructed as having a vertical or even past-vertical neck [14], [29]. The dichotomy of ‘upright’ versus ‘horizontal’ is not absolute. Even Giraffatitan has been given comparatively low-neck interpretations [39], [31], [32], [40]. But while some other sauropod taxa are reconstructed with descending necks at shoulders (e.g., Dicraeosaurus hansemanni [41], [42], [31]–[32], [43] and Nigersaurus taqueti [44]), it has recently been argued that all sauropods held their heads high [12], as will reviewed below. The presumption that many – perhaps most – sauropods held their heads high above ground level has become deeply entrenched and incorporated in derivative research regarding their physiology and behavior. Sauropod reconstructions are necessarily speculative, however, and likely have been subject to polarization, especially in the frequent absence of quantitative measurements (of slope and curvature) associated with the reconstructions.

Analysis of neck curvature in modern vertebrates provides a means to estimate neck curvature in sauropods. The starting point is to distinguish intrinsic curvature as that which remains in a vertebral column when all intervertebral joints are in an undeflected state. Osteological mounts of extant vertebrates are valuable resources illustrating intrinsic curvature and how it arises from their particular osteology. When the vertebral columns of extant birds, reptiles and mammals are assembled with the successive vertebrae spaced according to their separations in life, and with each joint undeflected, the columns assume a familiar and characteristic intrinsic curve associated with that taxon in an osteologically neutral pose (ONP). The process of estimating intrinsic curvature is of course neither absolute nor exact, nor is it immune to artistic bias and measurement error, in particular as regards speculation about the thickness of the intervertebral separation for extinct vertebrates.

**Figure 5:**
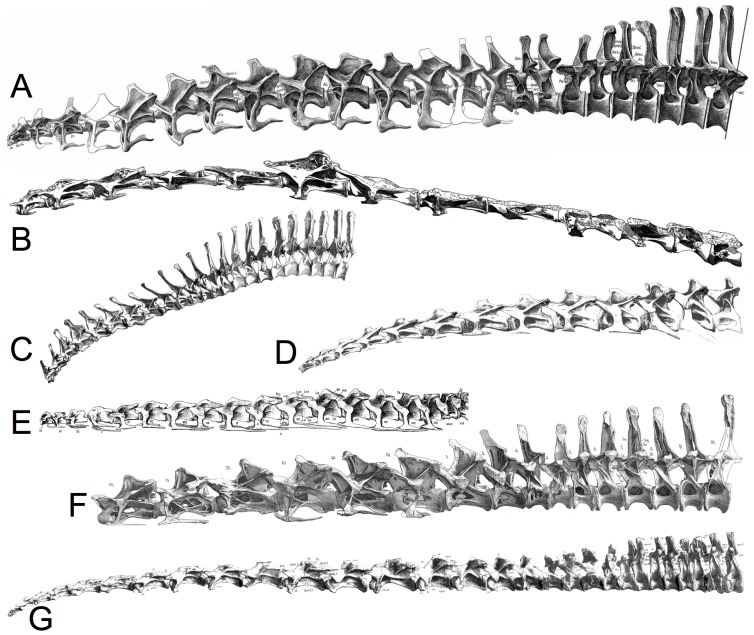
Estimation of sauropod ONP from illustrations. Composite figures are assembled into approximate ONP for partial or complete presacral columns for various sauropods: A: Apatosaurus louisae [45], B: Giraffatitan brancai [27], C: Dicraeosaurus hansemanni [86], D: Cetiosaurus oxoniensis, E: Euhelopus zdanskyi [4], F: Diplodocus carnegii [26], and G: Mamenchisaurus young [35]. Note that some exhibit a slight dorsal OIC cranially, and all are straight caudally. Cetiosaurus illustrations courtesy John Martin. The reconstructions are not to scale, however, the individual vertebrae within a column were adjusted as necessary to the same scale within each vertebral column [31], [32].

**Figure 6:**
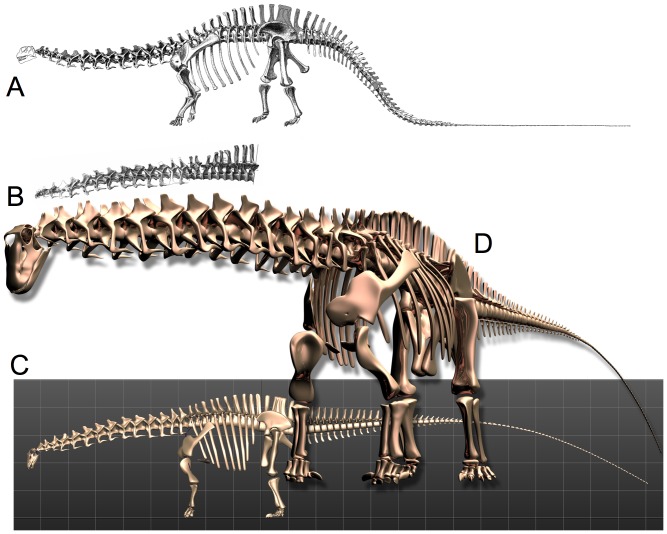
Revised Skeletal Reconstruction of Apatosaurus louisae. In the original 1936 reconstruction (A) of Apatosaurus louisae (CM 3018) [45] the pectoral girdles were positioned quite dorsally upon the ribcage, which created a downward slope to the anterior dorsal column at the shoulders, and hence a downward slope at the base of the neck. Reconstruction of the vertebral column from individual illustrations [B] corresponds closely to the skeletal illustration and was used as one check of the dimensional accuracy of a fully-articulated digital model of the specimen CM 3018 (C). All elements modeled individually to scale, based on archival sources [45] plus photographs and personal observation of the original material during its reassembly and remounting at Phil Fraley Studio, and scale orthographic drawings courtesy Philip Platt [pers. comm.]. In articulating and posing the digital model, the orientation and placement of the pectoral girdles and the angulation of the ribs incorporate many current contributions of studies of the articular skeleton, in particular the placement of the pectoral girdles [31]–[32, Phil Platt, pers. comm.].

Despite their common depiction with rising curvature at the base of the neck, reconstructions of the undeflected neck in sauropods in the cervico-dorsal vertebral columns suggest they were straight where the neck transitions into the anterior dorsals [30]–[32] (Figure 5). This basic finding is at odds with many depictions of sauropods, particularly brachiosaurids and camarasaurids, as will be discussed, but subsequent polarization of this work have summarily equated ‘straight’ (i.e., a lack of curvature) with ‘horizontal’ as: “When sauropod necks are reconstructed in ONP, their necks are horizontal” [17]. Straight, yes, but not necessarily horizontal. The goal of the 1999 study [30] was comparative neck flexibility, however, wherein Diplodocus sp. was found to be less flexible than Apatosaurus sp. when both were subject to the same criteria to limit intervertebral flexibility based on a modern avian model (see below), and both were less much flexible than the avian model. Head height of course varies trigonometrically with the slope and height of the base of the neck [31]–[32], and if the anterior dorsal column in diplodocids had sloped downward as originally depicted [25], [26], [45] that would have sent the neck on a downward slope as well (Figure 6a). But the modern interpretation of the pectoral girdles [cf. 32], [ 46] elevates the anterior dorsal column to approximately horizontal (Figure 6c, d), and this is naturally reflected in higher head heights. The ‘straight’ sauropod neck was subsequently reconstructed clearly horizontal or upward sloping, and when the less-than-avian 1999 estimates of diplodocid neck flexibility are applied to the revised bauplan, the 2005 studies [31]–[32] clearly showed that even the diplodocids could reach high enough that their feeding envelopes overlapped vertically with some other sympatric sauropod taxa regarded as ‘high browsers’. And yet these studies are persistently characterized as only suggesting these sauropods “… held their necks at or below horizontal, and could not raise their necks far above the horizontal” [12], [17], [47] and to have resulted in “low flexibility estimates” [48], promoting or perpetuating a false dichotomy, a polarization, between unnaturally-stiff-and-horizontal versus naturally-flexible-and-high-reaching.

### Depolarization

To depolarize the dichotomy between sauropod necks as straight-and-stiff-and-horizontal versus curved-and-flexible-and-upright requires replication and independent confirmation, and convergence of contributions from multiple directions. This will allow for more substantive pursuits, such as seeking deeper explanations for their extreme specializations. But proposals about sauropod neck curvature, pose, head height and so forth have been confounded and conflicted in the literature, and progress will likely require that they be understood in basically the following order:

1. intrinsic curvature of the vertebral column in the undeflected state.
2. intervertebral flexibility.
3. habitual pose for a variety of activities, including feeding, locomotion, and alert rest.
4. characteristic motions involved in browsing, drinking, display, surveillance.
5. vertical and lateral reach, feeding envelopes versus reachability volumes.

**Figure 7:**
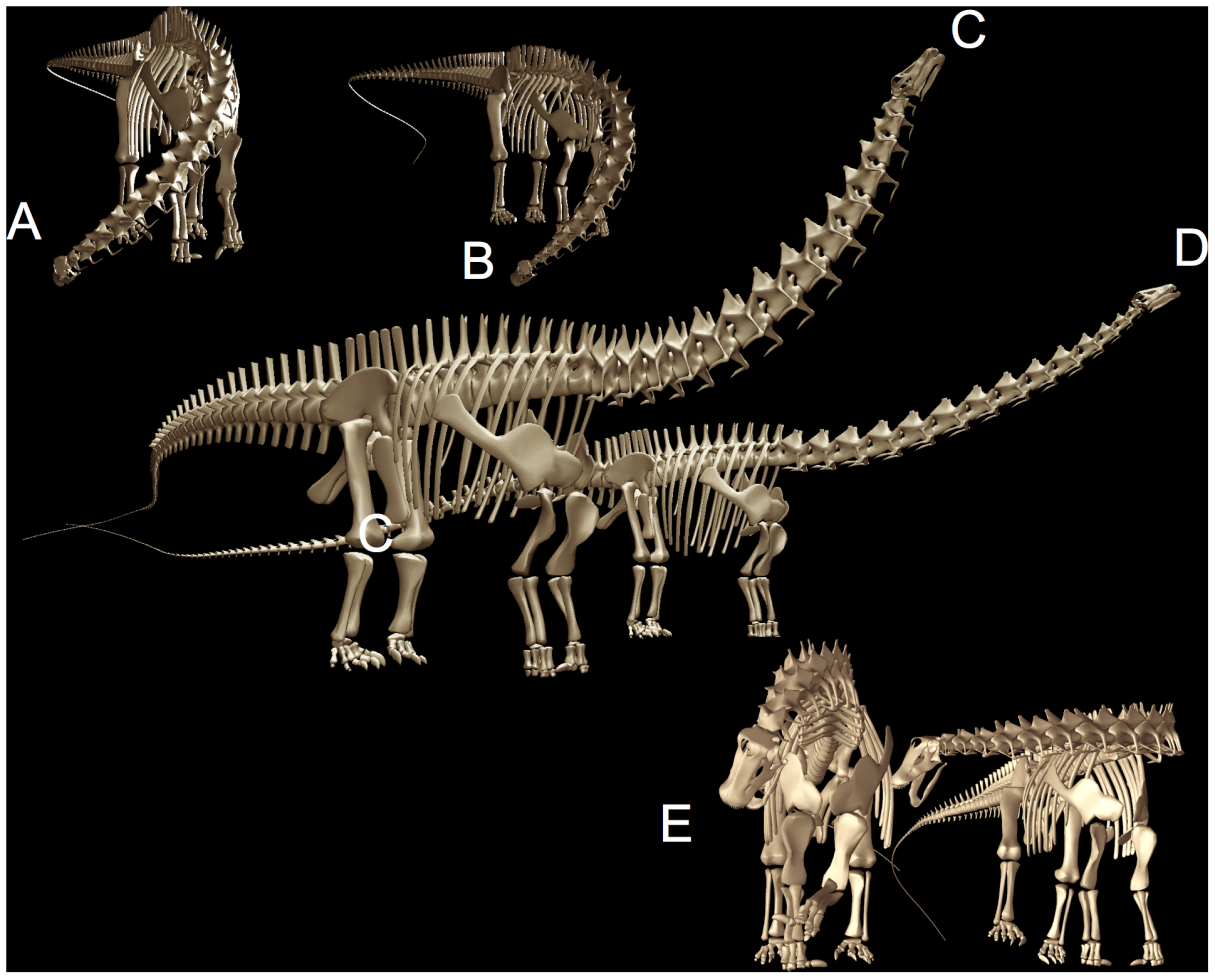
Diplodocids swept out a huge feeding surface, despite their relative inflexibility. Apatosaurus (A) and Diplodocus (B) are shown in extreme lateroventral flexion, reaching down and laterally to ground level, and in C and D in extremes of dorsal flexion (including dorsiflexion at the cranio-cervical joint) as if to reach as high as possible (see also overall feeding envelope visualization in Figure 20). Despite the enormous sweep of these necks, the vertebral joints, especially at the base of the neck of Diplodocus (C13 and C14) permit limited flexion prior to disarticulation (see Figure 9). While both necks sweep out a huge surface area, Apatosaurus, with its larger posterior cervical zygapophyses, could reach higher despite having a somewhat shorter neck than Diplodocus.

Just as the sauropod neck is becomes better understood in terms of topics (1–3), the post-cervical skeleton is as well, permitting refined estimations of the motions and characteristics of the vertebrate as whole. Importantly, studies of sauropod forelimbs and pectoral girdles [32], [49], [50] is resulting in the reconstruction of diplodocids and camarasaurids as much taller at the shoulder [31]–[32], [46] than when first described [26], [37], [45] (Figure 6). With their anterior dorsal columns no longer depicted as steeply descending, but instead horizontal or slightly rising through the shoulders, their heads would rise accordingly, and consequently even ‘low browsers’ such as Diplodocus could have engaged in an ecospace that many would consider as ‘high browsing’ [32] – see Figure 7. Camarasaurids and brachiosaurids were even taller at the shoulder absolutely, and had relatively longer forelimbs (compared to hindlimbs), resulting in even greater slope at the anterior dorsal column at the shoulder, and ultimately the slope of the base of the neck. Especially in the case of Giraffatitan brancai, one need not affix a swan-like neck for the head to rise far above that of the contemporaneous diplodocids [32]. Moreover, if much of the head elevation in the taller sauropods were achieved by leg elongation rather than neck curvature, they were still easily able to ‘high browse’ even if reaching down to feed, as modern giraffes often do today [51], [52], while reducing hemostatic pressures since the head would have usually been within a few meters of the height of the heart in ONP [H]-[C. Gunga, pers. comm., 53–55]. Differences in neck length and the slope at the base would differential head height and feeding specializations [56] would facilitate resource partitioning, without having to postulate that some had swan-shaped necks.

Estimates of head elevation as based on osteology alone is generally lower than that suggested by an analysis of forces and moments [57]–[60], and that is generally lower than the most extreme poses suggested on behavioral grounds [12]. Understanding of habitual neck postures will be refined and depolarized as better descriptions of the behavior of modern models are forwarded. At the time of this review, views on sauropod necks remain contentious primarily regarding topics (1–3), i.e., the intrinsic curvature of sauropod necks, their flexibility along their length, and especially, their pose at rest. In fact, much of the contention and polarization seems to reduce to whether these terrestrial giants held their heads swan-like or not when resting, which has little bearing on the important questions of sauropod biology, namely, how did they eat, rather than how they stood there when not eating.

## Methods

Inferences about sauropod necks (their curvature, flexibility, habitual poses, characteristic motions, and the relationship between the neck and the rest of the sauropod) rapidly lead from the hard evidence, the fossil material, to speculation. Even the reassembly of the undeflected vertebral column requires understanding how they were connected by soft tissues, and yet their intervertebral joints are not known and can only be inferred. Inferences about habitual neck posture and movement are even more derivative – and necessarily more speculative – as they build upon assumptions about intervertebral flexibility, which must build upon assumptions about the intervertebral joints, and so forth.

### Supporting Conjectures about Unpreserved Properties

The Extant Phylogenetic Bracket (EPB) [18], [19] is a method to support speculative inferences about properties that are not preserved in the fossil record such as a feature of soft tissue anatomy [19], [20], [61] or some aspect of behavior such as reproductive rate [62]). Consider some property P that is present in some taxa. Use P(t) to indicate that P is indeed present in taxon t. The EPB provides a means to support the inference P(t_0_) for some extinct taxon t_0_. Since P(t_0_) cannot be observed directly, an indirect argument is offered that involves an ‘osteological correlate’ O that reliably co-occurs with P in extant taxa and which is reliably preserved in the fossil record. O and P should be “causally associated” [18], [19], i.e., 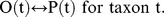



The co-occurrence of O and P is examined for the so-called extant phylogenetic bracket (EBP), namely the set of taxa that comprise the closest-related outgroup to the extinct taxon t_0_ [18]: 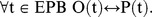



Given the physical correlate holds for extant taxa, the presence of the physical evidence O in a fossil of an extinct taxon t_0_ might be offered as evidence that this taxon also exhibited property P: 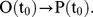



If the osteological correlate O is exhibited by all taxa in the EPB, then a so-called ‘Type I’ inference is supported for the extinct taxon, i.e., 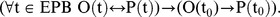



While some inference about the extinct taxon is well supported by observations on extant counterparts, the inference necessarily remains a speculation – just a “more constrained speculation” [19]. The strength of inference is weakened when the osteological correlation does not hold for all taxa in the extant outgroups, i.e., where some extant taxa that exhibit P but not C (or vice versa). Such counterexamples permit at best (‘Type II’ and ‘Type III’) inferences, with gradations [19], [20]. However, even in the absence of support by extant outgroups, a case may be based on a “sufficiently strong causal relationship” between C and P in extant taxa, i.e., “an argument of compelling morphological evidence[19], or ‘extrapolatory analysis’ [18]. A weaker, abbreviated form of this method, would rely on P occurring in the EPB, without support from an osteological correlate. This amounts to jumping to the conclusion without physical evidence: 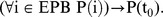



An invocation of EPB is particularly weak if, in addition to neglecting osteological correlates, draws conclusions based on only a limited sampling of extant taxa that exhibit the given property: 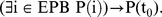



### Terminology

#### Osteologically Neutral Pose (ONP)

The undeflected state of an intervertebral joint, geometrically defined by centering the associated pre- and postzygapophyses.

#### Vertebral Axis

A vector constructed from cotyle center to condyle center, used to quantify curvature along a vertebral column (Figure 1).

#### Intervertebral Curvature

The angular difference between successive vertebral axes. Intervertebral curvature is zero when the axes are geometrically collinear (Figure 1).

#### Osteologically Induced Curvature (OIC)

The curvature of a vertebral column in ONP, as distinguished from curvature induced by joint deflection (Figure 1).

#### Range of Motion (ROM)

The set of all achievable combinations of mediolateral and dorsoventral flexion. As applied to a vertebral column: the set of poses (some subset of the product space of all individual joint ROM), also termed a ‘reachability envelope’.

#### Osteological Stops

Contact between vertebrae that limits angular deflection at a vertebral joint and provides load-bearing bracing against disarticulation. Osteological stops may be present independently for dorsiflexion and for mediolateral flexion, or not at all.

#### Zygapophyseal Safety Factor (ZSF)

During intervertebral joint flexion, displacement between pre- and postzygapophyses is limited by the surrounding ligamentous synovial capsule, which draws taut prior to their disarticulation, preserving a ‘safety factor’, a minimal overlap (typically 0.2–0.5 by lineal measurement). The ZSF provides a not-to-exceed limit on joint deflection, which is further restricted by soft tissues and behaviorally (see below).

#### Extant Phylogenetic Bracket (EPB)

To support speculation that some extinct taxon had some unpreserved property (e.g., a soft tissue structure or behavior) based on 1) observation of that property in closely-related living forms, the EPB, and 2) an ‘osteological correlate’. Presence of that correlated physical evidence in the extinct taxon supports inference that the unpreserved property was also present (see below).

### Estimating Intrinsic Curvature and Intervertebral Flexibility

The first two of the above five tasks concern estimation of intrinsic curvature and the extremes of what the joint geometry might allow – basic geometric (kinematic) aspects of sauropod neck shape and flexibility, and do not concern estimating their habitual poses, postural preferences, or behavioral tendencies. The success with which neck curvature and flexibility is replicated through the manipulation of the dry bones of extant vertebrates might be used to gauge the feasibility of estimating sauropod neck curvature and flexibility [12]. Clearly there would be little hope of learning about sauropod necks if extant vertebrates cannot be used as controls.

### The Osteologically Neutral Pose

Quantification of intrinsic curvature and joint flexibility requires first establishing the undeflected state of the intervertebral joints. The osteologically neutral pose or ONP [30]–[32] (Figure 1) defines the state of an deflected vertebral column, relative to which extremes of joint dorsiflexion, ventriflexion, and mediolateral flexion are subsequently measured [15], [63]–[65], [48]. Additional refinement to the operational definition of ONP is warranted, especially when the joint geometry suggests differing degrees of dorsal versus ventral flexibility, but a satisfactory convention is to define ONP as when the pre- and post- zygapophyses are centered and maximally overlapping, which often coincides at the centrum to parallel margins of synovial capsule surrounding the condyle-cotyle. ONP is not “merely the midpoint in the range of motion” as concluded by Taylor et al. [12] – vertebral joints are not equally flexible dorsally as ventrally. That is, flexibility is measured relative to ONP, not vice versa.

A vertebral column in ONP reveals the characteristic curve of the undeflected neck, which provides an important guide to how that neck is utilized [66], as discussed below. The characteristic curve of a vertebral column that remains when all joints are undeflected is termed here osteologically induced curvature (OIC), which medically correspond to regions of kyphotic versus lordotic curvature [67], and the anatomically-defined regions of the avian neck based on curvature and maximum dorsal and ventral flexibility [68]–[70], [64]. Determining the ONP of a sauropod's cervical vertebral column given only its bones requires is necessarily speculative since the cartilage, and thus the intervertebral spacing, is unknown.

### Accounting for Unpreserved Arthrology

Intervertebral joint flexion, in general, involves one vertebra rotating about an instantaneous or ‘momentary’ center of rotation relative to the other [71]. For vertebrates with amphiarthrotic amphiplatyan central articulations (such as lagomorphs, felids and humans), the instantaneous rotation center is not fixed, but rather, shifts depending upon the mechanical properties of the soft tissues and the instantaneous loading [72], [73]. Especially in those mammals such as lagomorphs whose cervical vertebrae are separated by a compressible nucleus pulposus, the resultant curvature of the column in life represents a reaction to all compressive, tensile and shearing forces imposed along the column, and a ‘dry bones’ articulation [12] would be expected to fail to predict either the column's curvature or flexibility in life. But static reconstruction has been used successfully to estimated pose and flexibility in vertebrates with diarthrotic central articulations, such as the closely-spaced heterocoelous vertebrae of birds [74]. Diarthrotic articulation involves the sliding translation of one surface upon a conformal, apposed surface, thus the instantaneous center of rotation is strongly constrained by their conformal geometries.

Wedge-shaped intervertebral disks contribute to the intrinsic lordotic or kyphotic spinal curvature of amphiplatyan vertebral columns, such as mammalian vertebrae and particularly apparent in the human lumbar spine [75]. Thick intervertebral disks are sometimes suggested to have formed some of the upward curvature in sauropod necks, where “… the thicker the disks were, the more upwardly flexed the neck was” [14], however the intervertebral disks in birds and reptiles do not have a nucleus pulposus and birds in particular are characterized by closely-spaced, conformal, diarthrotic articular facets [76].

**Figure 8:**
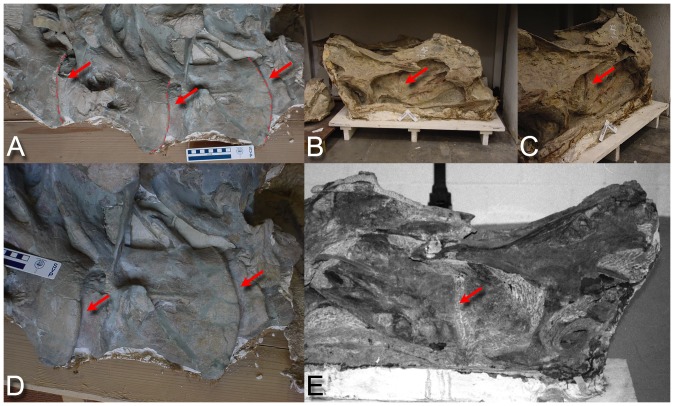
Sauropod intervertebral separations. Examples of articulated sauropod cervical columns with condyles deeply inserted into their associated cotyles, leaving intervertebral gaps of only a few centimeters (see arrows). Camarasaurus lentus (DNM 28, A and D) and Barosaurus (CM 11984, B, C, and E). Photographs by the author and J. Michael Parrish.

Numerous articulated sauropod cervical vertebral columns have been found with their central condyles deeply inserted within cotyles (e.g., see Figure 8, and Figures 16, 17, below). The preserved small intervertebral separations leave no room for the thick wedge-shaped cartilaginous pads that have been suggested might have curved the neck [14], nor should there they be expected. Moreover, the annulus fibrosus would unlikely shrink significantly due to its high density [77]. Instead of thick pads, intervertebral separations of a few centimeters between condyle and cotyle are suggested by their difference in radii of curvature (pers. obs.), which is consistent with the tightly-fitting central articulations found by Dzemski and Christian [15] in Ostrich (<1 mm) and Giraffe (7–9 mm), given that articular cartilage is negatively allometric with body mass [78].

### Estimating Intervertebral Flexibility

During joint flexion, the pronounced opisthocoely of sauropod cervical vertebrae greatly reduces uncertainty about the center of rotation, or the pivot point, about which they articulated. A cervical vertebrae can be regarded a rigid body comprised of three contact surfaces, the cotyle and plus paired postzygapophyses, moving as a unit in gliding contact over the surfaces of the condyle and paired prezygapophyses of the subsequent vertebra. As cotyle rotates over condyle, the postzygapophyses make gliding contact as they travel across their associated prezygapophyses (allowing for thin avascular layers of hyaline cartilage). Since both the central articulation and the zygapophyses are diarthrodial, with free sliding motion within their capsules, angular deflection at the centrum results in predominantly a translation or gliding motion of parallel articular surfaces, which is especially apparent as the postzygapophyses sliding across prezygapophyses. That translation must be arrested at some point otherwise disarticulation will occur. It is expected that sauropods, like modern vertebrates, arrested motion prior to disarticulation, preserving a residual overlap or zygapophyseal safety factor (ZSF).

Intervertebral joints flex dorsoventrally, mediolaterally, and in combination (dorsolaterally, etc.) to define a range of motion (ROM). Manipulation of dissections of turkey cervical columns [30] reveal that flexibility at each intervertebral joint is ultimately limited by the zygapophyseal capsules which prevent disarticulation by preserving a minimum overlap or zygapophyseal safety factor (ZSF). Manual exploration of the range of motion suggested that roughly 25–50% overlap (by lineal measurement) remained when the capsules were taut [30]. This was confirmed independently by a radiographic study of neck flexibility in ostrich which found that “bone would break before the zygapophyses would disarticulate” [M. Wedel, pers. comm, 79, 32]. Limiting neck flexion by preservation of zygapophyseal overlap was met with skepticism [80] due to the remarkable dorsal flexibility exhibited by camels [81], which actually does not require disarticulation [32]. Further confirmation of this safety factor is summarized by Dzemski and Christian [15]: “Extensive observations of living giraffes [63] and observations of living camels are in accordance with the data that were determined from the skeletons”. Moreover, the expectation that “… in vivo, muscles, ligaments, and fascia may have further limited movement” [30] has recently been supported [48] for the ostrich, however flexion in living birds approaches the limits of disarticulation [15]. While the ZSF predicts ‘best case’ estimations in extant vertebrates [30] (see also Figure 12), it's application may overestimate neck flexibility in sauropods with elongate tendonous cervical ribs [82].

**Figure 9:**
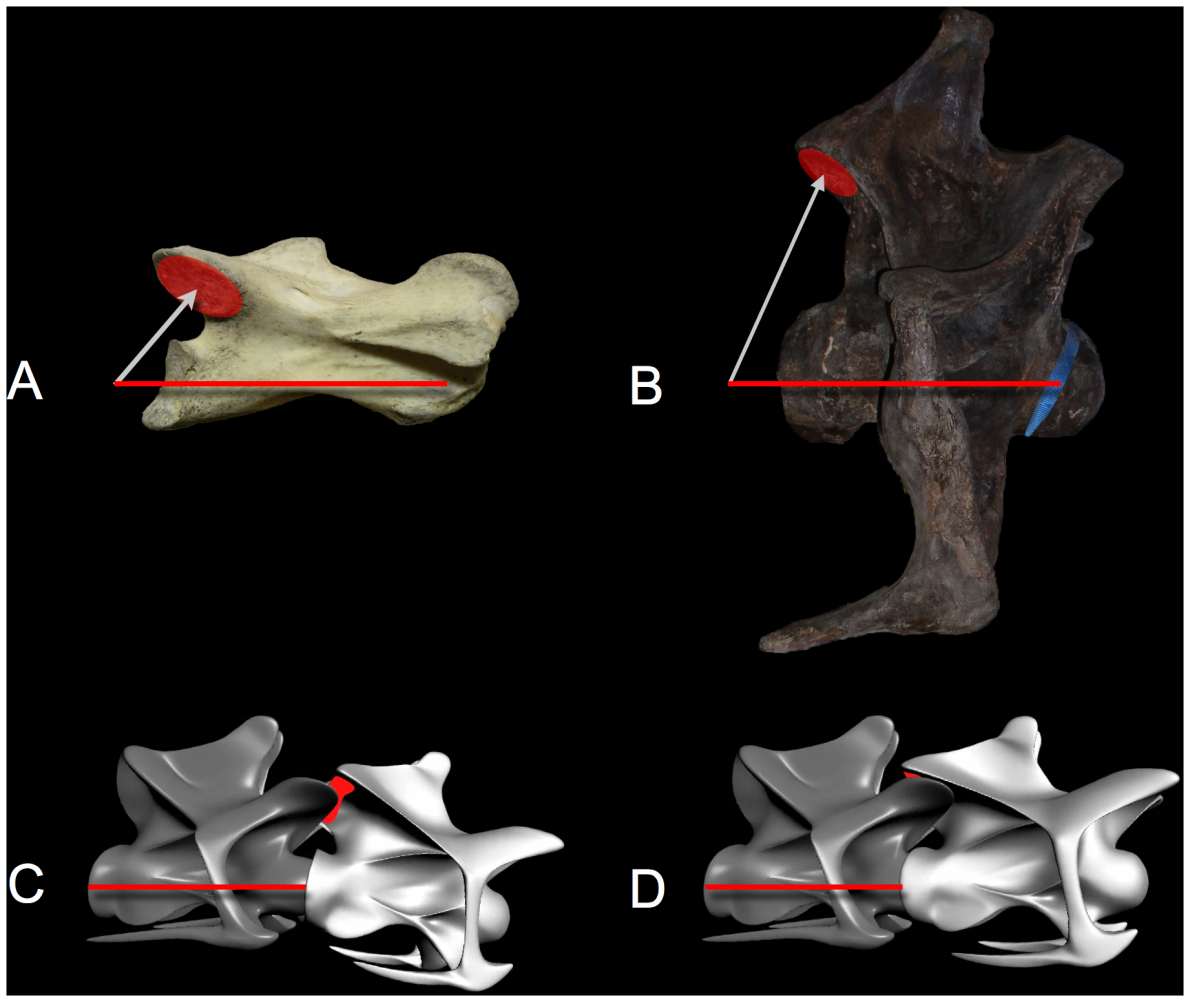
Sauropod necks did not have avian flexibility. Cervical vertebra C13 of the ostrich Struthio camelus (A) and C13 of Apatosaurus louisae (B) are scaled to equal vertebral axis length. The heterocoelous central articulation (A) and the opisthocoelous articulation (B), both have geometrically-defined centers of rotation defined by their centers of curvature in the sagittal plane. The ostrich postzygapophyses (red) are both relatively larger and closer to the center of rotation (white arrows) than those of the sauropod. The geometric consequence is that for any value of ZSF applied equally to the ostrich and to the sauropod, the former will have a greater range of motion. C and D show two articulated cervical vertebrae, C13 and C14, near the base of the neck of Diplodocus carnegii (CM 84) in maximum lateroventral flexion to the left (C) and maximum laterodorsal flexion (D), i.e., diagonal extremes of the range of motion. Note that the postzygapophyses (red regions) in C and D barely overlap their associated prezygapophyses (the ZSF is about 0.5). Struthio image courtesy John Martin; Apatosaurus image courtesy Virginia Tidwell. Supplemental material: Movie S1.

A conservative ZSF of 0.5 was used to estimate the relative neck flexibility in two diplodocids [30]. The relatively larger zygapophyseal surfaces in the posterior cervicals of Apatosaurus louisae permitting greater dorsal and ventral flexibility compared to the more slender counterparts in Diplodocus carnegii (Figure 7), but compare to the ostrich (Figure 9), the relatively small zygapophyses of Apatosaurus suggested far less than avian flexibility. The D. carnegii reaching laterally to harass A. louisae (Figure 7e) illustrates how at mid-neck any further lateral flexion would disarticulate its zygapophyses. Similar constraints apply to dorsiflexion, and will be discussed in the context of bracing the neck at the limits of head elevation.

### Osteological Bracing

**Figure 10:**
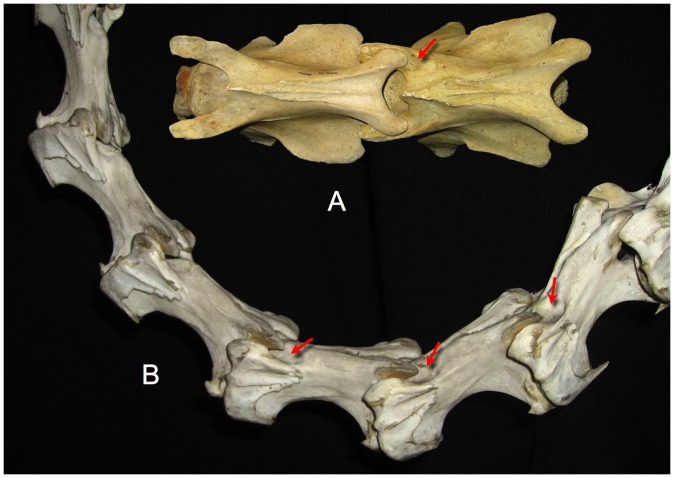
Osteological stops. The posterior cervicals of camel Camelus dromedarius (A) show pronounced depressions (see arrow) where the postzygapophyses make contact just posterior to the associated prezygapophyses with which they articulate. At the limit of travel in dorsiflexion the zygapophyses remain in overlap (contra [80]) and compression forces can be transmitted through the zygapophyses as the neck becomes effectively rigid and stable at the extremes of dorsiflexion. Pronounced osteological stops are also exhibited in many birds, such as the Greater Rhea Rhea americana (B, see arrows). Photographs by the author; rhea specimen at the Zoology Museum, University of Cambridge, access courtesy Matthew Lowe, and the camel vertebrae are at the Condon Museum, University of Oregon.

**Figure 11:**
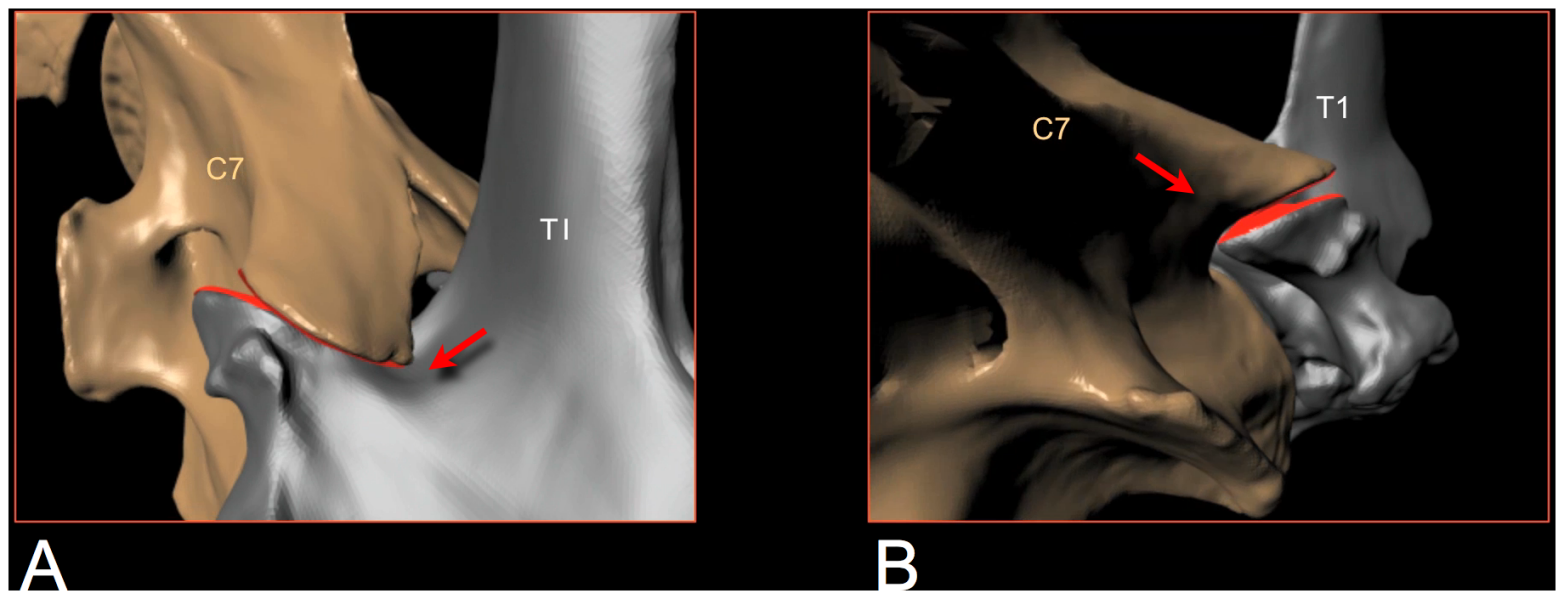
Bracing at the base of the giraffe's neck. The base of the giraffe's neck is braced to protect the intervertebral joints from excessive strain on their synovial capsules and to rigidify the neck as it reaches the limits of range of motion. As the neck is raised at the base (A), the postzygapophyses of C7 travel posteriorly until they wedge into depressions in the neural spines of T1 just behind the prezygapophyses (see arrow). Another bracing scheme applies when the neck is deflected laterally (B), In defecting the neck to the left, for example, C7 bears against the left postzygapophysis of T1, see arrow. In either dorsal or lateral flexion the two vertebrae progressively lock up firmly and stably. At these extremes the zygapophyses maintain substantial overlap (roughly a ZSF of roughly 0.5). CT data provided courtesy American Museum of Natural History and Timothy Rowe, University of Texas. Supplemental material: Movie S2, Movie S3.

**Figure 12:**
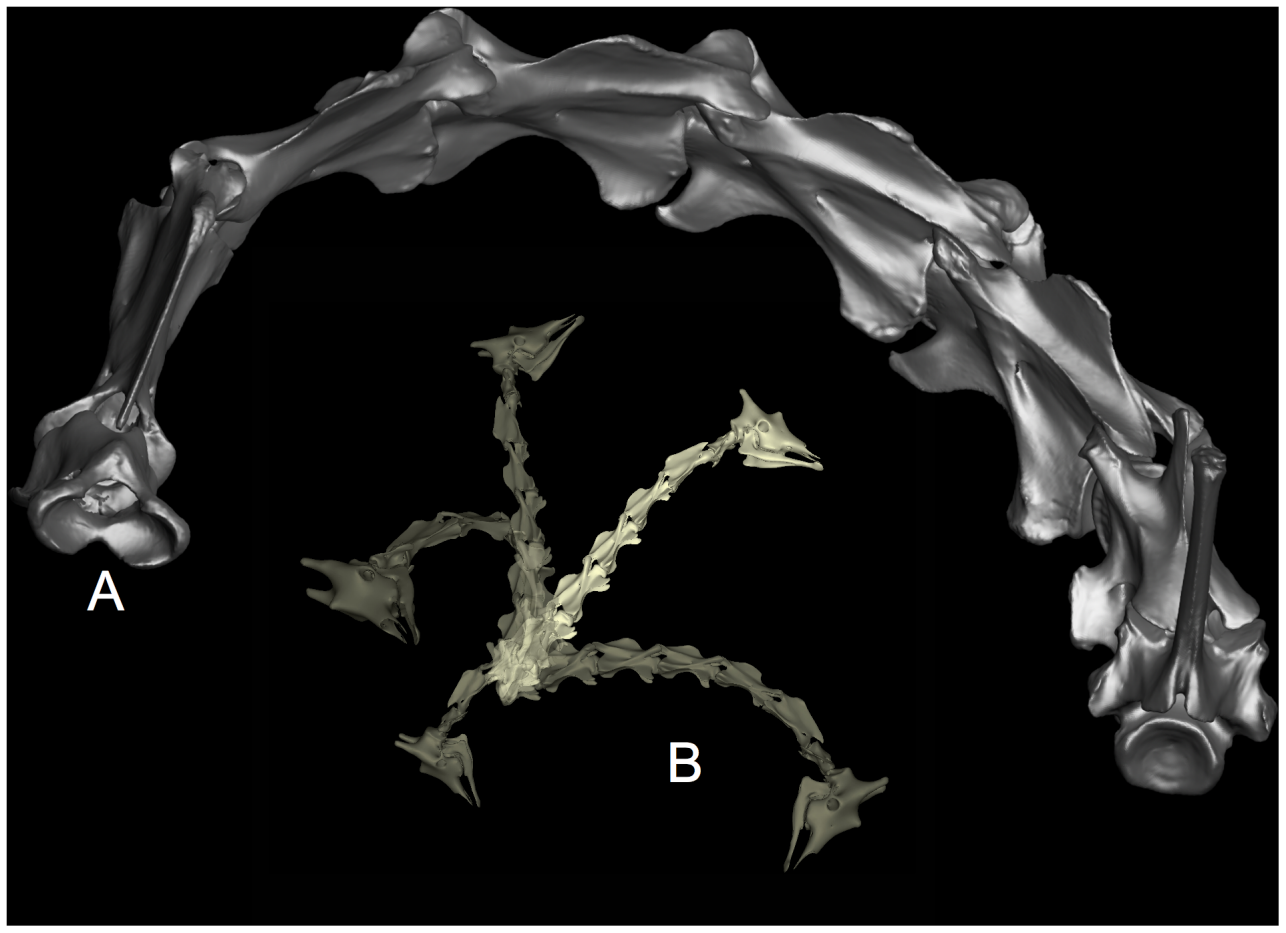
Giraffe flexibility is predicted by their joint geometry. The ability of a giraffe to reach vertically and to flex laterally to just reach its flanks is closely replicated by a digital model based on CT scan data of a recent giraffe (see also closeup in Figure 11). The zygapophyses remain in articulation with substantial overlap when they reach osteological stops at the base). CT data provided courtesy American Museum of Natural History and Timothy Rowe, University of Texas. Supplemental material: Movie S4.

In some vertebrates, in addition to limiting deflection by the ligamentous synovial capsule surrounding the zygapophyses, intervertebral joint flection may be limited by physical contact between vertebrae, e.g., between the postzygapophyses of one vertebra against the neural spine of the more caudal vertebra [32], [15], [48]. As dorsiflexion increases, for example, the postzygapophyses of one cervical vertebra may slide posteriorly until they fit neatly into depressions located just posterior to the associated prezygapophyses (pers. obs.; see Figure 10). Osteological stops for dorsiflexion are apparent in many birds, especially those with long necks, and in the base of the neck of large mammals such as giraffids, equids, and camelids. The prevalence of osteological stops in vertebrates is not well known, but it is noteworthy that they are clearly present in some vertebrates, and clearly absent in others (pers. obs.). Where they are present, experimental manipulation of vertebral pairs demonstrates that physical contact firmly braces the two vertebrae against further dorsiflexion (e.g., Figure 11) [15]. The neck ‘locks up’ and those vertebrae effectively becomes a rigid body protecting the intervertebral joint. Zygapophyseal bracing is also noted to be assist in stabilizing the neck against torsion and lateral tilting [83]. Osteological bracing may also prevent excessive mediolaterally flexion in some extant vertebrates (e.g., in the base of the neck in giraffes, Figures 11b, 12, and rhinos, pers. obs.).

It has been suggested that dorsiflexion in sauropods was also limited by osteological stops, given their presence in Camelus bactrianus, Giraffa Camelopardalis and Struthio camelus [15]. In an earlier study [30], the sauropod Diplodocus carnegii was estimated to have surprisingly little dorsal due to the relatively small zygapophyses at the base of the neck. Dzemski and Christian [15] rotated copies of Hatcher's [26] vertebra illustrations of the same specimen (D. carnegii, CM 84) from until the neural spines appeared to make contact in lateral view, as they do in ostriches. Their two-dimensional (2D) manipulation of illustrations, however, could not reveal that in three dimensions (3D) the zygapophyses would have been completely disarticulated far before the vertebrae would have contacted one another (Figure 9c, d). Dorsiflexion in Diplodocus (and likely in sauropods generally) was limited by the ZSF [30] without the additional bracing of bone against bone.

While some extant cervical vertebrae are braced ontologically as they reach the limits of dorsal or lateral deflection, many extant vertebrates do not exhibit apparent morphological adaptations (pers. obs.). Nor does osteological bracing against excessive dorsiflexion appear present in sauropods (although osteological contract may have braced the neck in extremes of lateral flexion in some sauropods [84]). In sauropod cervical vertebrae, three geometric factors argue against dorsal bracing by osteological stops: elevation of the prezygapophyses above the spinoprezygadiapophyseal laminae (sprl) [85], the ridge-like shape of the sprl, and the trajectory that the postzygapophyses would travel in dorsiflexion about the center of rotation at the centrum that would clear, rather than contact, these laminae. The sprl, which originates behind the prezygapophysis, ascends to the anterior aspect of the neural spine, and is ridge-like and devoid of a smooth depression or hollow to accept the loading by the postzygapophyses of the more anterior cervical vertebra during extreme dorsiflexion [30], [86], [4], [37], [38], [45]. Moreover, the prezygapophyses project anterodorsally relative to his lamina such that the postzygapophyses, pivoting about the central condyle, would not make contact with the sprl during its excursion posteriorly. Hence one cannot assume that sauropod vertebrae pivoted in a vertical plane until bone touched bone. Instead, dorsiflexion was likely limited by soft tissue constraints from the zygapophyseal capsule ligaments plus muscles and facia.

Due to their nearly spherical central condyles, sauropod intervertebral articulations can be regarded as universal joints of well-defined center of rotation and angular range of motion as imposed by limiting zygapophyseal displacement to preserve a safety factor (ZSF). Each successive pair of vertebral axes (Figure 1) defines a segment of a kinematic chain from base of the neck to the cranium. With each joint in an undeflected state (ONP), the chain forms a piecewise linear curve of characteristic form, such as the familiar sigmoidal shape in avian necks. The kinematic simplification of the neck to a chain of universal joints is adopted to many studies of neck flexibility [66], [15], . Since the centers of rotation are determined by the ball-and-socket geometry of the opisthocoelous central articulations, a ‘bare bones’ giraffe neck can be flexed to replicate observed limits flexibility by a combination of ZSF limit and osteological bracing (Figure 12).

### Osteologically-Induced Curvature

The normal division of human spine into regions of intrinsically lordotic or kyphotic spinal curvature arises partly by wedge-shaped intervertebral disks as mentioned, and partly by the vertebral osteology (as well as the posture assumed by an individual, of course). The osteological contribution can be subtle but accumulative, as in the slight wedge shape of the vertebrae in the lumbar spine [88]. It can also be dramatic: much of the the sharp elevation in the Giraffe neck is produced by the wedge-shaped osteology of the cervical vertebra at the base of the neck [89]; see Figure 1b, see also Figure 15b]. In general, centra that are shorter dorsally than ventrally, when articulated and aligned in ONP form a natural upward bend, such as common at the base of the neck in many extant birds and reptiles. This curvature is not due to flexion; is ‘in the bones’ but may be further accentuated by dorsiflexion, of course. Vertebral centra that are shorter dorsally than ventrally will produce dorsal OIC, typically at the base of the neck, which serves to elevate the head. Combinations of these morphologies along the cervical column produces a variety of intrinsic curves in ONP.

**Figure 13:**
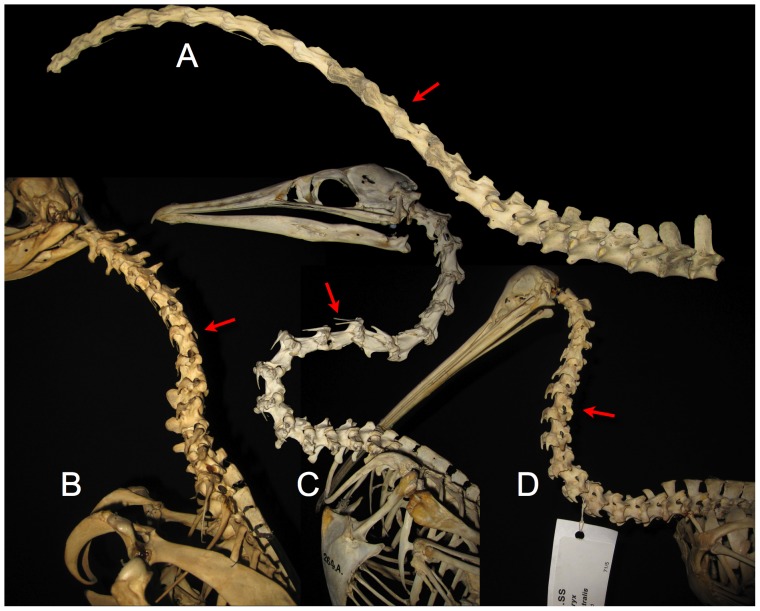
ONP for various birds. The avian neck has a sigmoidal curve that is formed intrinsically by its osteology when the vertebrae are articulated in ONP. The alert resting head height for the ostrich Struthio camelus (top) is higher than predicted by ONP [15] (and the ostrich often further retracts the head during locomotion [98]). Many other birds, however, do assume a pose close to ONP as their characteristic alert resting posture: Cape Penguin Spheniscus demersus (bottom left), Flightless Cormorant Phalacrocorax harrisi (middle), and Kiwi Apteryx australis (bottom right). Note inflection points (arrows). Photographs by the author and John Martin; specimens at the Zoology Museum, University of Cambridge, access courtesy Matthew Lowe.

**Figure 14:**
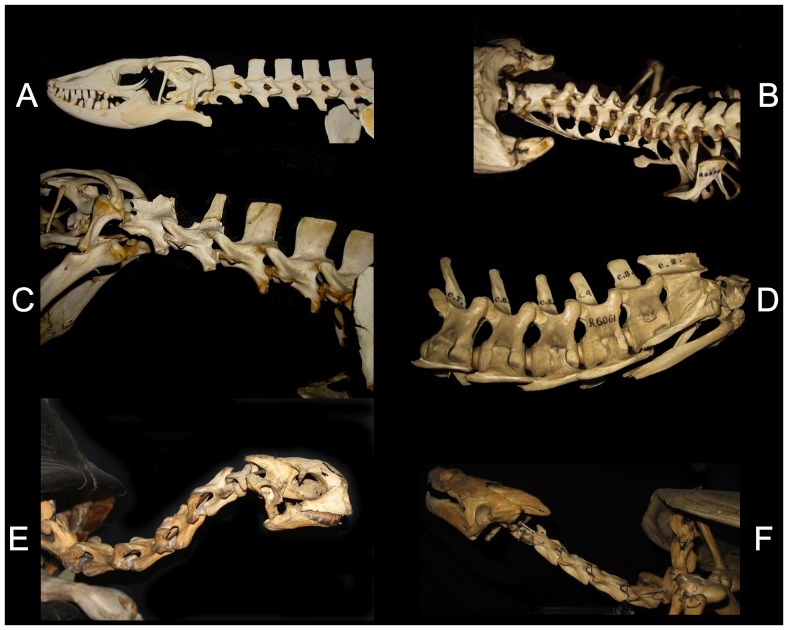
ONP for various reptiles. The Nile Monitor Varanus nilotictus (A) and Komodo Dragon Varanus komodoensis (C) have very straight necks in ONP. Head elevation, if any, is primarily through the slope of the anterior dorsals. In contrast, the crocodilians Alligator mississippiensis (B) and Crocodylus acutus (D) have gently rising necks in ONP. The Seychelles tortoise Testudo elephantina (E) has an inflection in curvature; note that its characteristic head elevation arises in ONP. The cryptodiran snapping turtle Chelydra serpentine (F) curves monotonically from a vertical descent caudally to nearly straight cranially. Photographs by the author and John Martin of specimens at the Zoology Museum, University of Cambridge, access courtesy Matthew Lowe.

**Figure 15:**
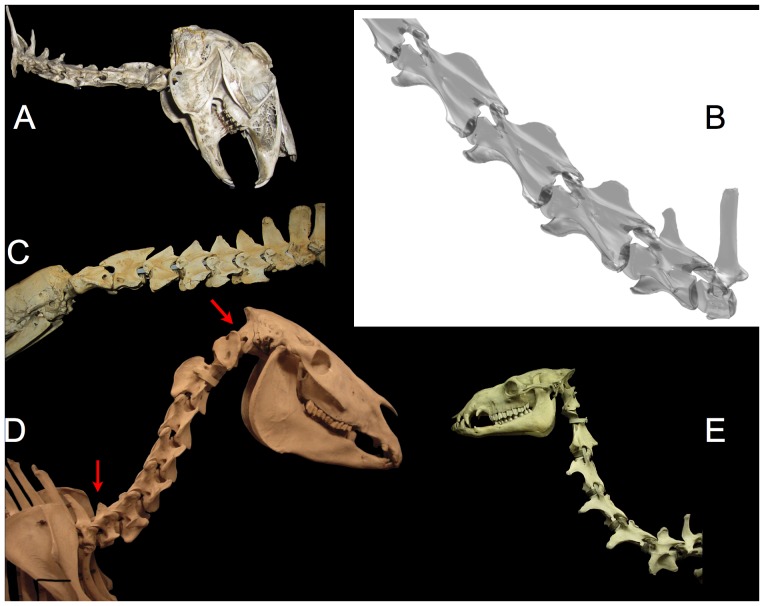
ONP for various mammals. Mammals have more or less dorsally-curved necks that tend to raise the head intrinsically. ONP is characteristic of mammals in alert rest and locomotion (an exception is exemplified by the Brown Hare Lepus europaeus (A) which assumes ONP for locomotion and exploratory behavior [97: fig. 17-3] but not in alert rest [95], [96]. The giraffe Giraffa camelopardalis naturally rises in ONP (B, note the deep insertion of condyles within cotyles consistent with dissections [15]), and assumes approximately this pose in locomotion and alert rest [63], [89], [92], [93]. The nearly straight necks of the Giant Anteater Myrmecophaga tridactyla (C), also mounted in ONP, is characteristic of habitual alert resting pose of alert rest, locomotion pose and feeding. The horse Equus caballus (D) and camel Camelus dromedarius (E) also hold their heads close to ONP in alert rest and locomotion. Note that the cranio-cervical joint is undeflected (arrow) as well as the entire cervical column. Photographs by the author (camel photograph by J. Michael Parrish); hare, anteater and horse specimens at the Zoology Museum, University of Cambridge, access courtesy Matthew Lowe. The camel is at the Field Museum of Natural History. The giraffe is a 3D digital model placed in ONP based on CT data courtesy American Museum of Natural History.

Many birds also have ventral OIC cranially, which together with the rise at the base of the neck, creates a sigmoid curve with an inflection in curvature mid-neck (Figure 13). Reptile necks generally form simpler curves, from varying from nearly straight in most lacertilians (Figures 14a, c) [90], [91], to more elevated with a more or less pronounced built-in arc in crocodilians (Figures 14b, d) [94; pers. obs]; and turtle necks in ONP position the head in a characteristic pose, that is steeply descending caudally then rising cranially, sometimes with a sigmoidal curve in ONP (Figures 14e, f). Mammal cervical vertebral also form a simple arc-like curve (Figure 15), from nearly straight in anteaters and hares (Figures 15a, c) to more substantially curved in giraffes, horses, and camels (Figures 15b, d, f). In mammals the built-in dorsal curvature is greatest at the base and diminishes cranially. The sigmoidal shape characteristic of horse necks, incidentally, is little reflected in the osteology, but superficially by the epaxial musculature. The catenary shape of the camel neck derives from the descending slope of the anterior thoracic vertebrae combined with the dorsally curved cervical column. The underlying osteology of the mammalian cervical column is not ‘S’ shaped nor ‘U’ shaped but ‘J’ shaped, and to the extent there is an inflection point in curvature, it is not within the neck, but at the atlantoccipital joint. Like the letter ‘J’, the column begins with high curvature which diminishes as the curve ascends.

### Behaviorally-Induced Curvature

Caution is needed to distinguish between behaviorally-induced curvature and that which is intrinsic to the osteology, particularly when attempting to draw broad generalizations about default behavioral postures [12]. Neck posture varies with activity [15], [63]–[64], from alert rest to locomotion and feeding, and vertebrates do not all assume a similar strategy for holding their head in alert rest (discussed below). The ONP provides a baseline relative to which characteristic poses for resting, locomotion and feeding can be described. The ONP corresponds to the alert rest pose in at least some birds and reptiles [95]–[97], and while yet to be systematically studied across mammals, ONP predicts the default alert head height for large herbivorous mammals at alert rest and in locomotion [98, pers. obs.]; Camel and Giraffe [15], [63]–[64] often hold their heads slightly higher that predicted by ONP). Some mammals (e.g., felids and lagomorphs [95]) can assume a ‘sphinx-like’ pose by retracting their heads sufficiently to balance over their shoulders when resting (discussed further below). Others cannot, but have alternative means of minimizing energy expenditure. Also, while ONP may predict a default alert pose for birds in general, there are exceptions. Ratites such as the Ostrich (but not the Cassowary or Kiwi, pers. obs. and Figure 13d) hold their heads far above the height predicted by ONP [15], [63]–[64].

Speculation regarding the relationship between ONP and characteristic poses of the neck during rest and locomotion for sauropods seems of less importance to understanding their biology than how they used their necks for feeding. The relationship between ONP and the characteristic pose for feeding in modern herbivores is straightforward only for grazers, while many low browsers will take advantage of vegetation that requires raising the head above ONP, and high browsers will very frequently feed by ventriflexion far below ONP [99]–[103].

### Skeletal Reconstructions: Illustrations, Mounts, and Models

Given the rarity and inaccessibility of physical mounts of dinosaur skeletons, skeletal drawings and composites have traditionally used to reconstruct and subsequently analyze sauropod vertebral columns. More recently, 3D digital models are being used in preference to relying on 2D artwork [31]–[32], [104], [87]. Regardless the medium, all such reference material is subject to issues of restoring missing or damaged vertebrae. Unfortunately, the illustrations and physical mounts which are frequently relied upon as primary sources of information about sauropod osteology are subject to subtle yet significant alterations. Digital 3D modeling and articulation of scan data brings with it new as well as old problems of subjectivity.

### Illustrations

Skeletal illustrations have been relied upon to both summarize the bauplan of a given taxon of sauropod, and as source material on which to base estimates of head height and speculations regarding feeding, and so forth. Significant artistic liberties are sometimes noted [31], [32] but usually dismissed as either within the realm of possibility, or as artwork. Nonetheless illustrations are often trusted as authoritative.

**Figure 16:**
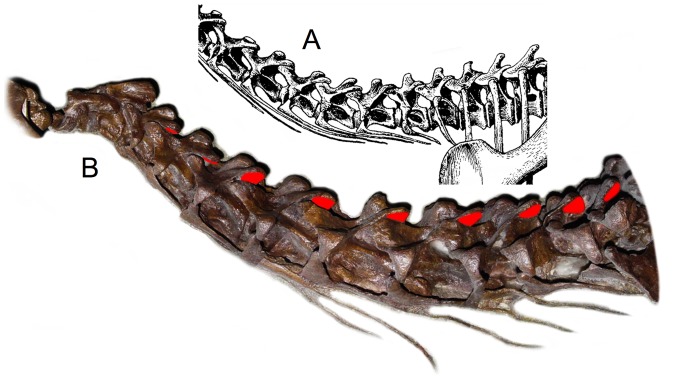
*Camarasaurus* had a swan neck taphonomically, but not in life. The 1925 skeletal reconstruction of the juvenile Camarasaurus [38] (A) accurately replicates the curvature of the neck as found (B), but the zygapophyses are illustrated misleadingly as if they were aligned, in ONP, suggesting that the upward curve is intrinsic and ‘built in’. The original specimen, however, is obviously contorted into a dramatic opisthotonic pose, with the zygapophyses disarticulated throughout much of the neck. Red indicates the exposed postzygapophyses (compare with nearly identical opisthotonic pose in the larger specimen USNM 13786-310D, Figure 17). Disregard for this extreme opisthotonic distortion in subsequent skeletal depictions, some portraying the neck comfortably achieving a near vertical pose [14], [29] has resulted in a nearly universal expectation that Camarasaurus had a natural swan-like curve to the neck. Photograph of Camarasaurus lentus CM 11338, by the author.

The macronarian Camarasaurus, for example, is usually depicted to have had a sharply rising neck at the base, largely due to illustrations based on the juvenile C. lentus CM 11338. The original specimen was preserved in a severe opisthotonic posture, with the cervicals wrenched back and the zygapophyses displaced out of articulation (Figure 16a). The skeletal illustration (Figure 16b) [38: plate XVII] however, shows the neck with the same curvature but with the zygapophyses drawn as if aligned, in ONP, suggesting that the steep neck curve was intrinsic [38], [14], [29], likely contributed to the widespread current presumption that this sauropod had a natural swan-neck. The same depiction of death-as-life pose arises in Wiman's 1929 [4] illustration of Euhelopus zdanskyi in life (Figure 2) with an ascending neck drawn with precisely the same curve as when it was found, in an opisthotonic state. And as mentioned, Janensch's [27] illustration of the skeleton of Giraffatitan brancai (Figure 4b) depicts a steeply-ascending neck, seemingly in ONP, which, bears little resemblance to the actual fossil material in the cervico-dorsal region [105] (Figure 4a); while the neural spines were not preserved at the base of the neck, the centra were found in articulation, with central articulations approximately in ONP. In the skeletal reconstruction, however, the cervico-dorsal centra acquired a wedge shape and the neural spines are figured with aligned zygapophyses, suggesting this neck ascended in ONP. The slope subsequently been exaggerated to vertically (or past vertically) [29: fig. 20.7], however some skeletal reconstructions show the cervico-dorsal region [39], [32] as close to the straight.

**Figure 17:**
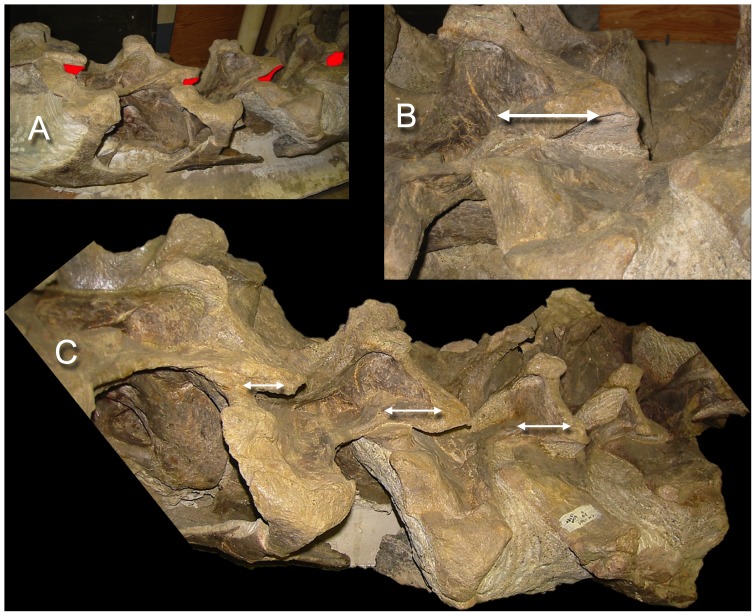
Another *Camarasaurus lentus* in opisthotonic pose. A partly-prepared block, USNM 13786-310D, reveals a ‘death pose’ with curvature very close to that of the more familiar juvenile specimen CM 11338 (Figure 16). In both specimens the postmortem dorsiflexion disarticulated the zygapophyses such that it was preserved in a pose that was unlikely attainable in life. Red indicates exposed postzygapophyses, and the white line segments indicate the extreme displacement of the zygapophyseal pairs from ONP. Photographs by the author.

While skeletal reconstructions may incorporate artistic liberties, some degree of independent verification is afforded by the detailed steel engravings or photographs of the individual vertebrae illustrations were published in the original descriptions by C. Gilmore, J.B. Hatcher, W. Janensch, W. Wiman and others. These illustrations can be scanned, composited, and placed into articulation to roughly reconstruct their ONP (Figure 5) [31]–[32]. But 2D illustrations are of limited use, as they collapse or obscure aspects of the 3D structure that are essential in understanding their articulation, as evidenced with problems that arise in estimating flexibility, even in the case of pure dorsoventral movement from lateral views [15].

### Sculpted Inaccuracies in Skeletal Mounts

The neck of the iconic Giraffatitan brancai mount (Figure 3) is reconstructed with its neck in ONP. The cervico-dorsal vertebrae are extensively restored, since the neural arches of the original specimens were missing. The restored vertebrae were sculpted in such a manner as to make them appear to bend naturally, with all zygapophyses aligned, centra wedge-shaped, and even the anterior and posterior margins of the condyles and cotyles made to appear undeflected in that steep curve. Not only are the missing vertebral arches fabricated to form a steeply-ascending neck, the centra are curved to follow that bend, in marked contrast to with Janensch's illustrations [105] of the original material (c.f. Figures 3, 4e). Although only the centra were preserved in the block from C10 through D2, those vertebrae were found in articulation as a very straight column based on their collinear ventral margins, and the ridges of condyle and cotyles were parallel indicating that they were roughly in ONP. The historic and familiar swan neck of the mounted skeleton, while impressive, is a fabrication.

While extreme in the case of the Berlin mount, it is not uncommon for neural spines to be restored in sauropod skeletal mounts as if they were in ONP. The Apatosaurus ajax at the Yale Peabody Museum (YPM VP 001980), for instance, has a gently-curved sigmoidal-shaped neck. Close inspection shows that the zygapophyses are centered, as if the vertebrae were in ONP. Still closer inspection, requiring a ladder to reach up and tap on the darkly-varnished plaster, reveals an artistic amalgam of real material and plaster (pers. obs.). The gracefully-ascending curve to the neck appears to have been conceived first, then the details of the restoration made to neatly fit that vision.

The neck of Camarasaurus lentus USNM 13786-310D (Figure 17) was preserved in articulation in a pronounced opisthotonic pose (‘death pose’). The vertebrae were dorsiflexed to the extent that the zygapophyses were disarticulated, as was the case in the juvenile C. lentus CM 1133 (c.f. Figures 16 and 17). This extreme state of dorsiflexion is again likely beyond what could have been achieved in life, given that degree of disarticulation. USNM 13786-319D (originally CM 11373) was used as reference for a sculpted replica for public display (M.K. Brett-Surman, pers. comm.), the opisthotonic neck curvature was accurately replicated, however the zygapophyses were sculpted as centered, as if the neck curvature were intrinsic, not due to extreme dorsiflexion. The displayed sculpture further reinforces the incorrect expectation that Camarasaurus had a steeply elevated neck at the base.

**Figure 18:**
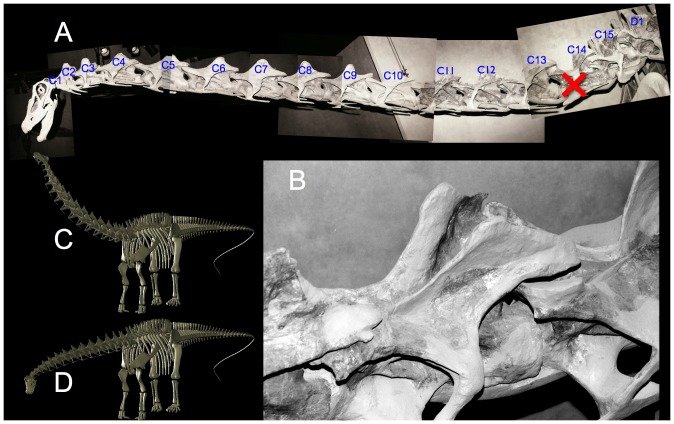
This *Diplodocus* has a false kink in the neck. The Denver Museum of Nature and Science mount of Diplodocus longus DNMS 1494 has a sharp upward bend that appears intrinsic since the vertebrae in the vicinity of C13–C15 appear undeflected. The curvature, however, is an artifact of the restoration of the fragmentary neural spines, and not exhibited by any other diplodocid specimen including the Carnegie Museum of Natural History Diplodocus carnegii CM 84, a cast of the first 10 cervicals of which were used for the Denver mount. Those cervicals caudal to C10 are heavily restored and induced the misleading suggestion of an upturned neck. Taylor, Wedel and Naish [12] claim that “… computerized studies are not as objective as they may appear, since seemingly Stevens and Parrish could not replicate the flexibility of actual specimens” presuming that the entire neck of DMNS 1494. In fact, the flexibility estimates from [30] would have permitted the head to have reached such heights (see [32] and C). The specimen they refer to (A) has a sculpted bend that is not representative of other, more complete specimens of Diplodocus that emerged straight from the shoulders (D). Photographs by the author, access courtesy Kenneth Carpenter. Supplemental material: Figure S1, Figure S2.

The Denver Museum of Nature and Science Diplodocus longus DMNS 1494 is also mounted with an upward bend in the neck at its base (Figure 18). Again, the vertebrae appear undeflected with zygapophyses in neutral alignment indicating that the bend is intrinsic to the neck. While the vertebra from C1–C10 are based on a cast of Diplodocus carnegii CM 84 (Kenneth Carpenter, pers. comm.), those posterior to C10 have restored neural spines, and the sculpting required to integrate the zygapophyses had set them into position as if the vertebra were undeflected, thereby suggesting a sharp intrinsic bend around C13–C14. The neural spine restorations are built up around the placement of the zygapophyses. The induced kink in the neck is inconsistent with other Diplodocus material, including the original CM 84 posterior cervical vertebrae. Unfortunately, a subsequent study trusted the Denver mount as osteologically accurate [12].

### Models

2D skeletal illustrations provide only limited insight into vertebral articulation, but have been resorted to given limited access to, and manipulation of, original specimens, especially those that are mounted. Yet entire articulated vertebral columns can be manipulated in a virtual 3D space provided their morphology is converted to digital form. Fossil vertebrae can be digitized to capture their surface morphology and subsequent digital retrodeformation [106] can remove at least some of the postdepositional distortion that would otherwise preclude their re-articulation. The problem of reconstructing missing (not merely distorted) elements remains, however. Filling in for a missing vertebra by duplicating then scaling an adjacent vertebra is not unheard of, but clearly of greater aesthetic than scientific utility.

**Figure 19:**
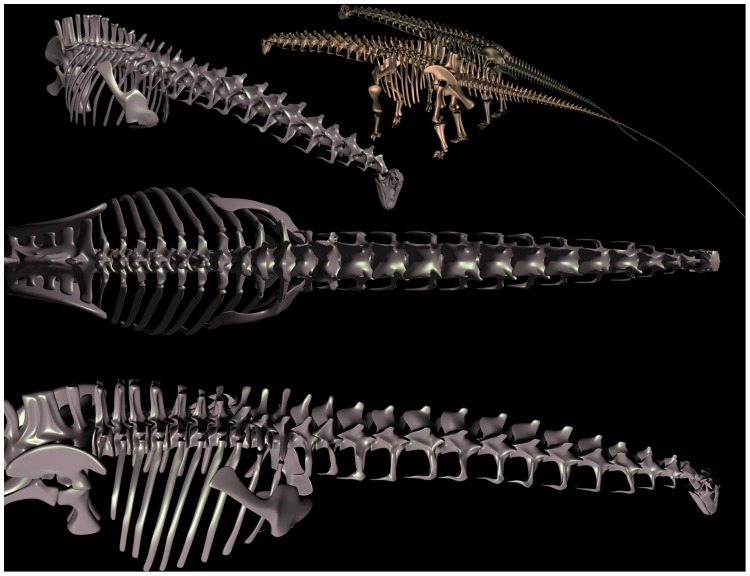
Details of the digital modeling of *Apatosaurus louisae*. Archosaur vertebral morphology varies smoothly along the axial skeleton, and the gradual changes from one vertebra to the next is amenable digital modeling by ‘blend shapes’ (see text regarding digital modeling). Through a multi-step process, first deformable generic forms are created for all elements then used to create specific variations on that shep. For example, a generic dorsal rib is constructed, then several specific ribs are modeled to match the corresponding original fossil material, with the remaining intervening elements created by interpolation, and finally each element is painstakingly sculpted and adjusted to capture individualities of the original specimen such as the irregularities in the cervical ribs, compared to the original specimen (Figure 5). The process of creating a digital scale model, like sculpting in a more conventional physical medium, shares the same goals of faithfully replicating the morphology and dimensions of the original. Like physical sculptures, it is a matter of judgment as to when the resemblance is sufficient, and as to what is to be regarded as artifactual, such as an apparent distortion due to preservation. Unlike physical sculptures, these models are readily edited and successively refined, and most importantly, readily articulated without need for a physical armatures. As a visualization tool, digital models greatly facilitate the appreciation of design as the bauplan emerges from the aggregation of the component pieces (note that A. louisae is accompanied by a Camarasaurus lentus, to scale).

An alternative to digitization of original material is to begin with a set of deformable 3D models can be subsequently formed to closely resemble the morphology of the original specimens. Deformable 3D surfaces can be created using subdivision surface model techniques [107], and using blend shape animation techniques [108], [109], adjusted to match dimensional data from multiple sources (effectively lofting 3D surfaces to match 2D profiles derived from archival images and illustrations, or when available, 3D data from surface scans, etc.). The models can be interpolated by creating a model that is an interpolate of two such 3D shapes, creating a more accurate restorations in cases where the original reference fossil material is missing, inaccurately restored, or intractably distorted (Figure 19) – see below.

**Figure 20:**
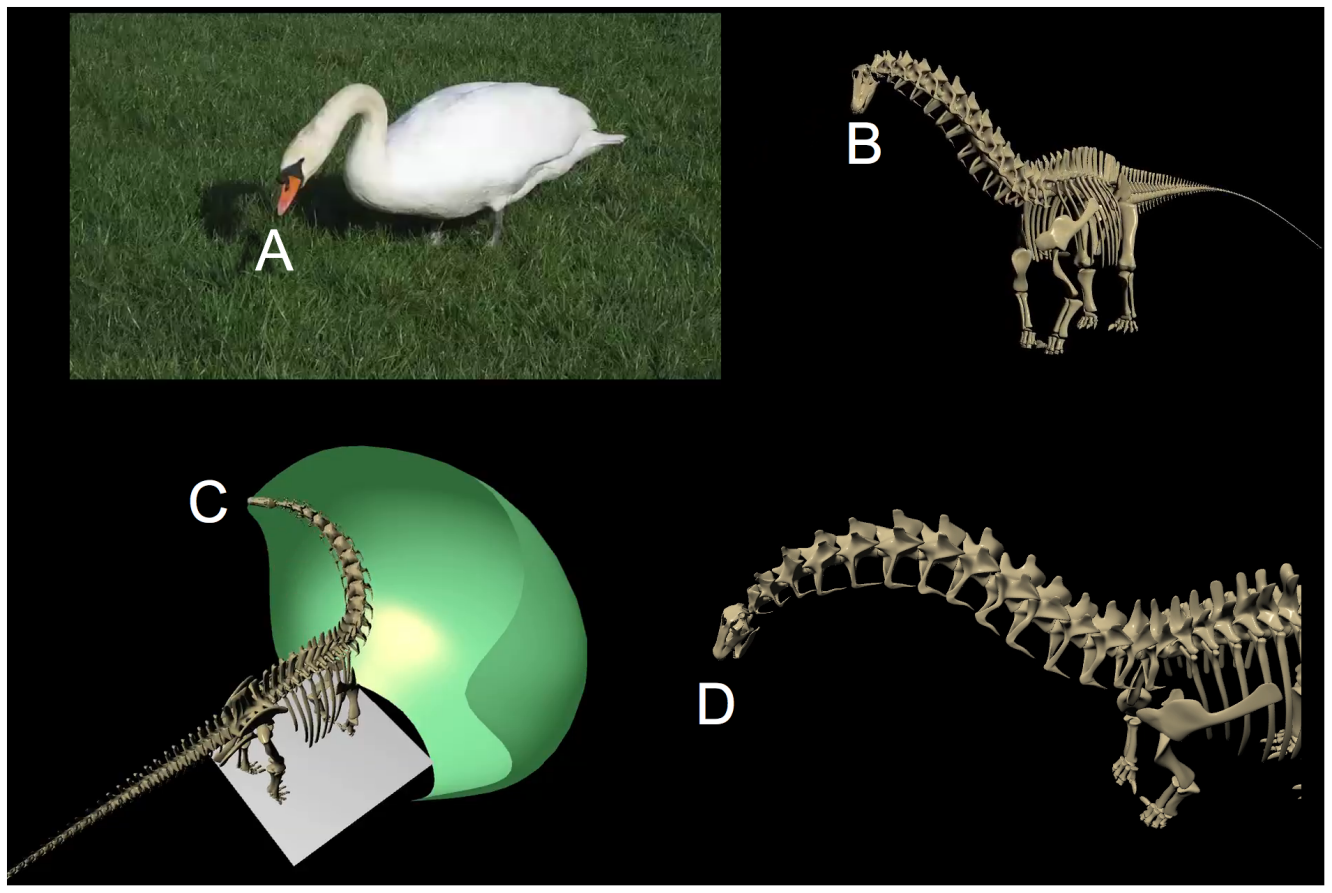
Long necks, but not swan necks. In addition to sweeping out a broad ‘feeding envelope’ (a curved surface of maximum reach [28]), sauropod necks are sometimes expected to be able to pull the head back to reach closer to the animal to explore the volume within this surface, (e.g., [113: fig. 12.1]), somewhat in the manner of a swan (A). While Apatosaurus could place its head at any point across an enormous feeding surface (C), the neck was not able to retract the head back towards the body (B, D). Supplemental material: Movie S5, Movie S6, Movie S7, Movie S8.

Through a laborious process of building then adjusting generic models of axial and appendicular elements to fit specimens, eventually entire articulated digital skeletons can be constructed (Figures 6, 7, 20, 22, 23) that approximate the shape and dimensions of the available reference material, faithfully replicating that morphology which is judged undistorted while attempting to correct for distortions, defects, and missing elements in the source material. Doubtless, subtle artistic license can be introduced in the digital sculpting process, just has it has been known to happen with plaster or pencil. Just as the term ‘sculpting’ may connote an artistic and often subjective process, so too is digitally-sculpted modeling. But then a digitized specimen is a model as well, and reflects subjectivity and artistic bias as a result of the many steps including decimation, filtering, and smoothing to fit a satisfactorily smooth surface that approximates the original surface prior to digitization, followed by artful manual correction of voids, registration errors, and under-sampled regions. Retrodeformation necessarily introduces subjectivity as well, e.g., in further adjusting a model to remove scaling artifacts induced by an automated retrodeformation process [106].

**Figure 21:**
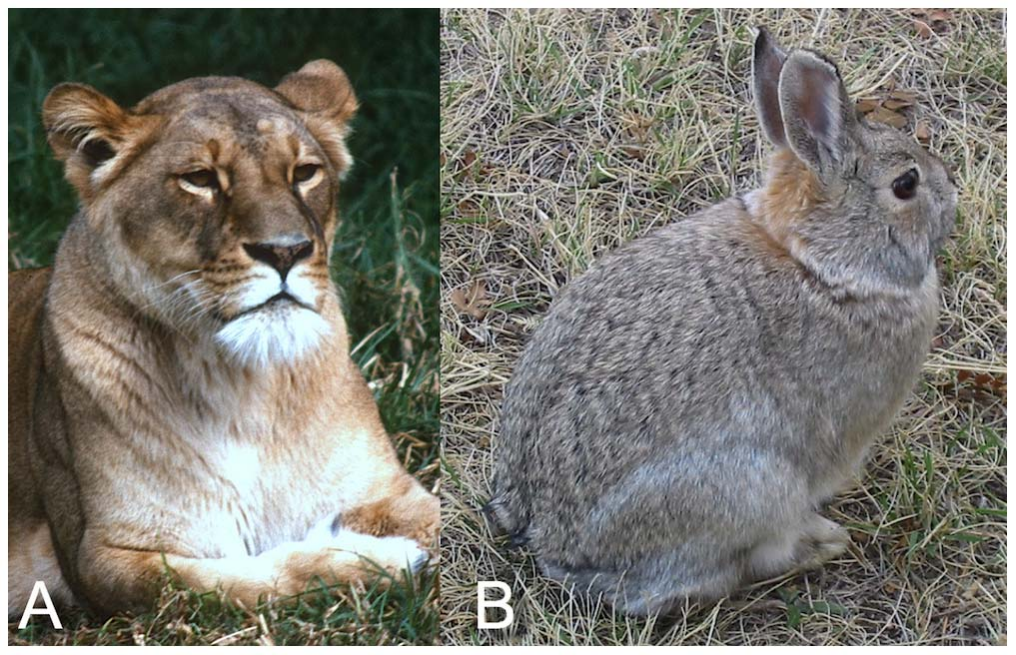
Some mammals relax in an alert posture by retracting their heads over their shoulders, but most do not. The sphinx-like alert resting posture in Panthera leo (A) and Sylvilagus nuttallii (B) is achieved by maximum dorsiflexion at the base of the neck (C7-T1) and maximum ventriflexion at the head to keep the head level, as shown by radiographic studies (95–97). But few mammals can achieve this feat. Most quadrupeds hold their heads cantilevered before the shoulders with the intervertebral joints in a relaxed ONP posture and the weight of the head and neck carried by dorsal ligaments and muscles. The horse, for instance, holds its head high in alert rest (as in Figure 15d), with all joints of the cervical column, including the cranio-cervical joint and the C7-T1 junction undeflected, in ONP. Photos by the author.

**Figure 22:**
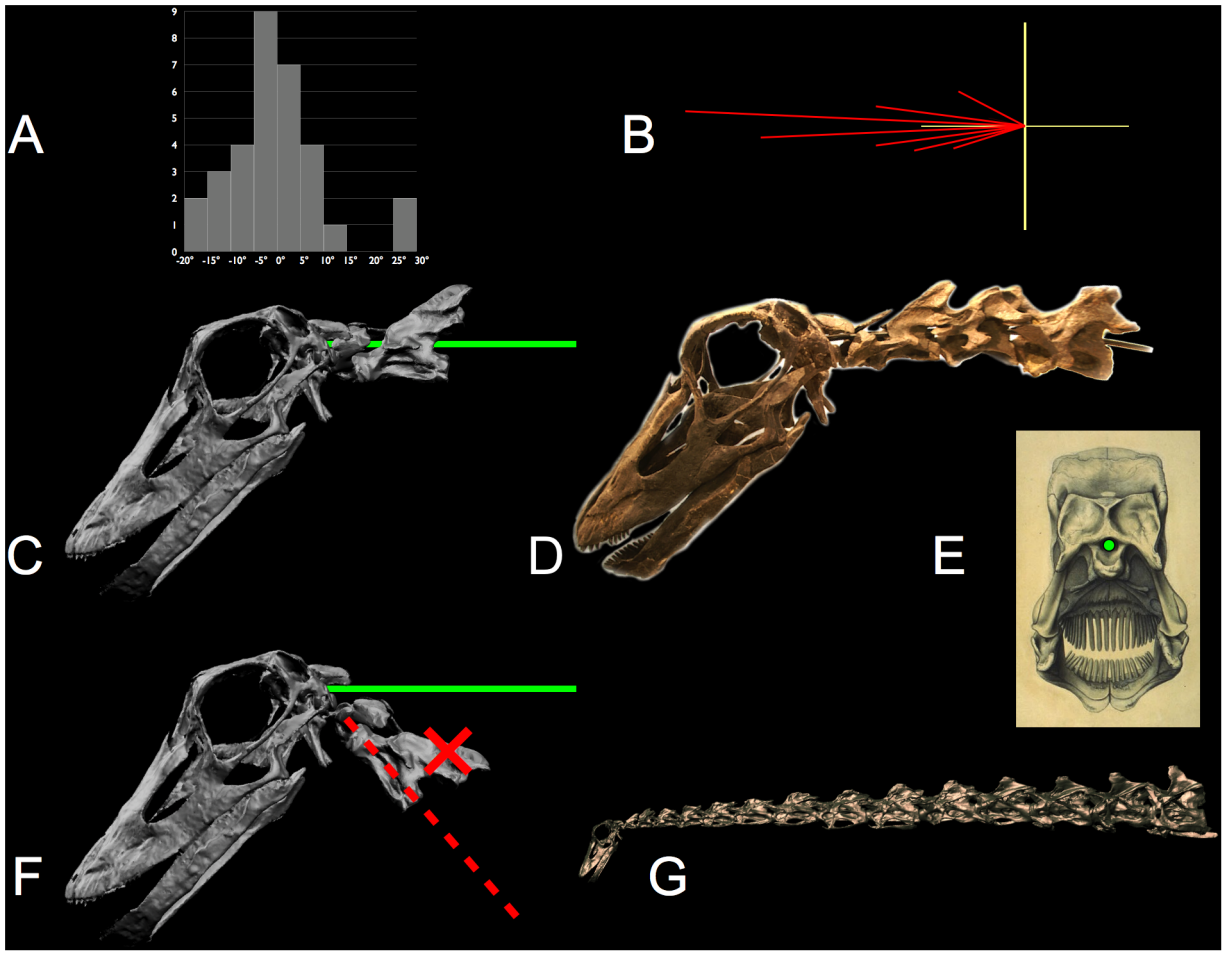
Inner ear orientation is consistent with subhorizontal sauropod necks. The lateral semicircular canal (LSC) is approximately horizontal in alert birds. The orientation α (see text) is plotted for 32 species of birds [111: fig 7a] as a conventional histogram (A) and polar histogram (B) with 5° intervals (c.f. expanded-scale plot in [132: fig. 2]). When a sauropod cranium is similarly oriented (α=+5°), the rostrum slopes downward (by −15° in Camarasaurus lentus and by −37° in Diplodocus longus) [44], [135]. The LSC also constrains the slope of the neck cranially. The neural canal passing through the atlas-axis is collinear with the foramen magnum, as illustrated by the solid green line in C and the physical armature in the original specimen (D) of Kaatedocus siberi, SMA 0004 [133] – see also the location of the foramen magnum (indicated in green) in the posterior view (E) of Diplodocus [134]. Consequently, with the cranium oriented relative to gravity as indicated by the LSC, and with the cranio-cervical joint undeflected, the anterior neck is roughly horizontal [44]. Taylor et al. [12], however, misinterpreting the anatomy, suggest “… the foramen magnum and occipital condyle are [both] at a right angle relative to the long axis of the skull …” so that the atlas-axis inserts posteroventrally to the cranium, and consequently they falsely conclude the anterior neck ascends steeply as indicated by the red dashed line in F, from [12: fig. 4]; they figured an even steeper neck for Camarasaurus. But properly interpreted, the anatomy of the occiput, the atlas-axis, and the LSC, together with observations of habitual head orientating in the EPB, supports the interpretation that the necks were habitually subhorizontal cranially in diplodocids (E) and camarasaurids (as depicted in Figure 23) [44]. The digital reconstruction (C, F, and G) is based on data courtesy Andreas Christian and Gordon Dzemski. Photo (D) by the author. Supplemental material: Movie S9. A turntable movie depicting the spinal cord (red) entering the foramen magnum of Kaatedocus siberi.

**Figure 23:**
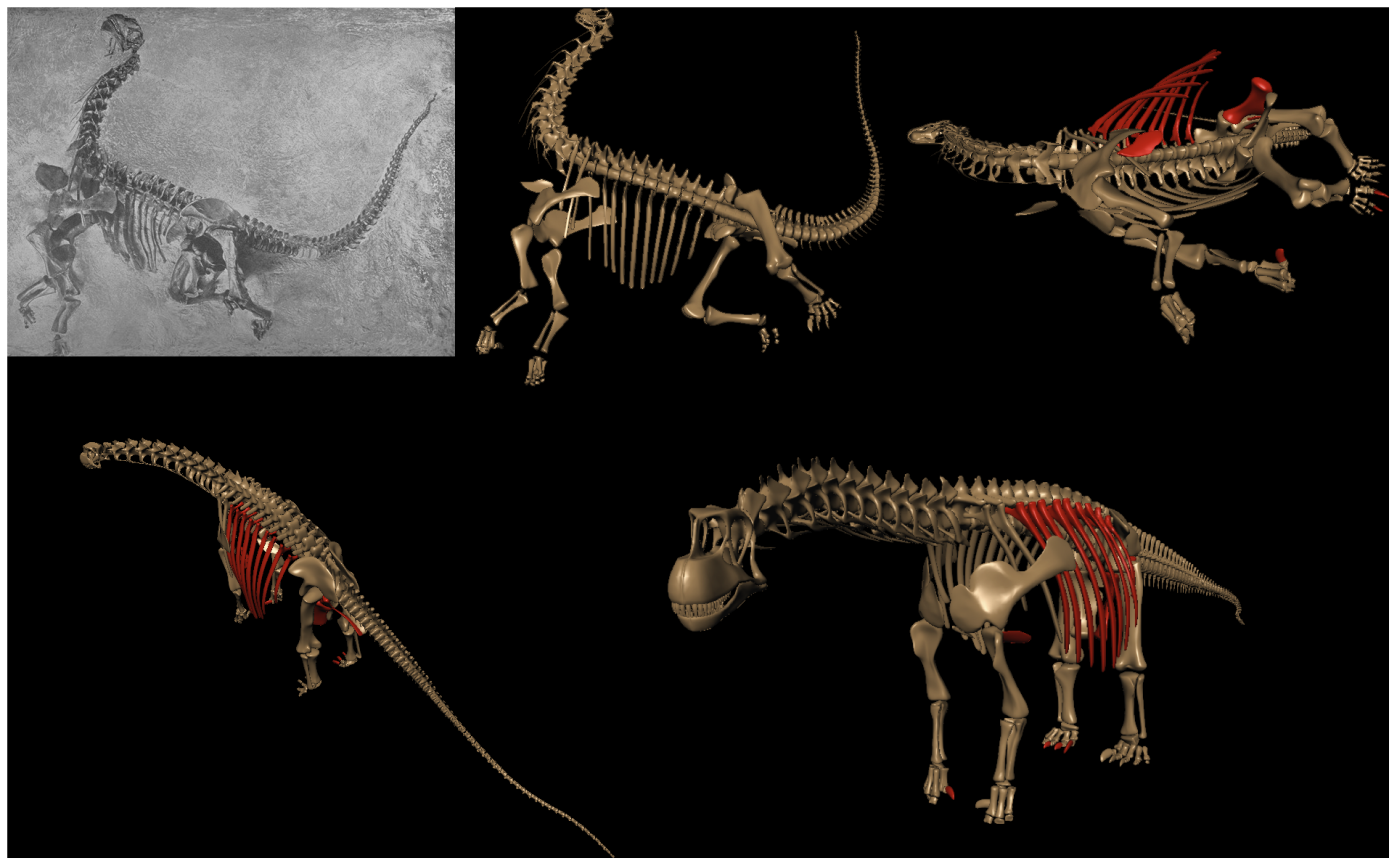
Resurrection of a juvenile *Camarasaurus lentus*. The iconic swan-like ascending neck of Camarasaurus sp. [38] likely derives from the opisthotonic pose of the remarkably complete specimen CM 11338 (upper left). However, when all elements are modeled individually and placed into ONP, the opisthotonic pose in the neck and the axial twist through the dorsal column is removed revealing that this sauropod had a rather short neck that extends straight from the anterior dorsals, which raised the neck with a slight incline (see also [136]). Red indicates elements that were missing in the original specimen. This model was created for the Carnegie Museum of Natural History, with cranial modeling contributed by Scott Ernst, forelimb modeled with reference to digitization data of AMNH 664 and scapula coracoid of CM 11338, both courtesy Ray Wilhite (see below regarding digital modeling). Supplemental material: Movie S10. Animation of Camarasaurus from its death pose into a life pose near ONP.

## Results

The following general inferences regarding sauropod vertebral joints appear supported by the EPB, with avia and reptilia as outgroups:

1. Intervertebral central articulations were diarthrotic, with close intervertebral separations.
2. Anterior cervical vertebrae were essentially straight (negligible OIC in ONP).
3. Posterior cervical vertebra had slight ventral OIC in ONP
4. Intervertebral joint flexibility was limited by synovial capsules surrounding the zygapophyses which draw taut prior to permitting disarticulation, preserving a ZSF.
5. Cervical vertebrae were limited in dorsiflexion by the ZSF, not by osteological stops.

Specific conclusions regarding sauropod necks would include the need to revise the reconstructions of brachiosaurids and camarasaurids based on 1–3, above. Regarding 4 and 5, above, the relatively ‘stiff’ necks of sauropods (by avian, but not reptilian, standards) and their kinematics suggest a coherent role for the sauropods neck with regard to feeding. While not yet explored in detail, the following provides a review that draws on the deep understanding of neck function in extant vertebrates towards better understanding the corresponding function of sauropod necks. As discussed below, some of the insight derives not by analogy with birds, but by how the analogy with birds fails, and yet resembles that of another, very distant group: browsing mammalian herbivores.

### Necks for Sweeping Out a Surface Versus Necks for Exploring a Volume

While the relatively inflexibility of sauropod necks compared to birds is sometimes viewed with skepticism [80], [12], the kinematic implication of relatively small zygapophyseal facets (compared to their distance from the center of rotation) is clear: less angular deflection is permitted prior to their disarticulation (c.f. Figures 9). The greater intervertebral flexibility in avian intervertebral joints permits birds a greater behavioral repertoire than that of those vertebrates with stiffer, straighter necks. Kinematically, the redundancy in the avian head-neck system permits control of both the placement and orientation of the head within a volume [65], by adjusting all cervical joints to form a smooth spline-like curve that “… behaves effectively as a (pre-shaped) flexible rod that, given the orientation and position of the two endpoints, takes the shape that minimizes the bending energy” [66]. The avian neck divides into regions that can work individually or together to explore a large volume in three dimensions, e.g., for preening and selective feeding [110], [111], [65] (Figure 20a). In contrast to the avian sigmoidal curve, the monotonically-curved necks of lacertilians, crocodilians and mammals is simpler kinematically, with the head neck system operating primarily to direct the head in two dimensions by flexion at the two extremes of the column [112], [95]–[97].

The cervical column may be regarded kinematically as a spline-like chain of fixed-length links between joints of limited angular mediolateral and dorsoventral flexibility [66]. Consider first a neck that is initially completely straight. Flexion near the base of the neck reorients the distal vertebral column and results in the head sweeping across a curved surface or envelope centered upon the base of the neck. This constitutes the ‘reachability envelope’ of the neck (Figure 20c). Flexion cranially reorients the head on a smaller radius of curvature. Uniform flexion along the entire column produces an arc-like curve which indeed reduces the radial distance (the chord) from base to head but the tangent at the head is also affected. To maintain the head pointing in a given direction while retracting it back towards the base of the neck requires different segments of the column working in opposition, effectively creating a sigmoidal spline curve. The built-in sigmoidal curve in the avian cervical column permits its joint flexibility to be distributed relative to that curve, which facilitates independently controlling both the pointing direction of the head and the position of the head [66], [110], [111]. In contrast, a simple monotonically-curved neck in ONP must create an inflection point by dorsiflexing caudally and ventriflexing cranially (Figure 20b, d). Consequently, while mammals can achieve wide reachability envelopes (turning to point directly behind themselves [114]), and reptiles generally less so, they must strain to retract their head even moderately, often choosing instead to take a step back.

Consider the consequences of varying vertebral length, count, and flexibility, either singly or in combination. First, increasing vertebral length alone increases reach linearly and the surface area of the reachability envelope quadratically. Increasing intervertebral flexibility, particularly caudally, also increases surface area roughly linearly (and both vertebral elongation and specialized flexibility is apparent in giraffes). Next, while holding overall neck length constant, increasing vertebral count while trading off intervertebral flexibility and vertebral length off can produces a tradeoff, resulting in the same reachability envelope. But increasing vertebral count without proportionately reducing intervertebral flexibility greatly dramatically increases the kinematic redundancy of the neck [66], and hence its repertoire of postures. Further increasing intervertebral flexibility compounds this increase in the space of possible neck configurations. Long-necked birds such as the swan and ostrich have done just that, with considerable intervertebral flexibility at each of 20 or more joints. In contrast, sauropod specialization has tended towards generally towards increases in vertebral length and count but not flexibility, suggesting that their necks were specialized for other tasks than those to which birds use their necks: for sweeping across a surface, not for exploring a volume.

### Speculation About the Habitual Resting Pose in Sauropods

Taylor, Wedel, and Naish [12], [17] argue that sauropods habitually held their heads high. With annotations Ci added in the following quotation for subsequent reference, they claim [17]: “*A substantial literature on extant amniotes (mammals, turtles, squamates, crocodilians and birds) shows that*:

C0: *“living animals do not habitually maintain their necks in ONP. Instead …*
C1: *“the neck is maximally extended at the cervico-dorsal junction*
C2: *“and maximally flexed at the cranial-cervical junction*
C3: *“so that the mid-cervical region is near vertical*.
C4: *“This is true even in apparently short-necked animals*. …
C5: *“The fact that elevated, extended necks are widespread across Amniota means that*
C6: *“elevated necks should be assumed for sauropods in the absence of evidence to the contrary*.
C7: “*Elevated neck postures for sauropods are indicated by the extant phylogenetic brackets at the levels of Saurischia, Archiosauria, Diapsida, Reptilia, and Amniota*.

Recall that the EPB method supports a Type I inference about an unpreserved property P in some extinct taxon t_0_ if a physical property O is identified, that is correlated with P in all members of the extant outgroups comprising the EPB, and O is present in traces of the extinct taxon: 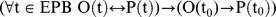
and if no physical evidence 0 is offered then the method would degenerate to simply asserting that if the property is apparent now, it was then: 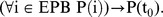



Regarding the conjectured C0–C4 [12], [17], the property P is a behavior combining maximal dorsiflexion at the base of the neck and maximal ventriflexion at the head. This behavior would contort the neck far from ONP, but being ephemeral, would not be expected to have left a trace in the fossil record. Claiming that all extant amniotes assume this pose in alert rest (the validity of which is addressed momentarily), they argue that this behavior should also be assumed of sauropods (C6). While specifying extant phylogenetic brackets (C7), they offer no osteological correlates for the behavior they attribute to sauropods, nor a ‘compelling morphological evidence’ [19]. They propose a behavior of sauropods simply on the basis of the (purported) ubiquity of that behavior across Amniota. For EPB support, they cite a radiographic study of the resting posture of various laboratory animals (monkey, cat, rabbit, guinea pig, rat, chicken, lizard, and frog) [95], plus two follow-on studies [96]–[97]. Indeed the mammals (rat, guinea pig, rabbit, cat, and monkey) do habitually rest in an alert state, however that same study showed that the non-mammalian subjects did not assume such an extreme posture: “… in lizard and frog, the cervical column was held near earth horizontal, when animals were in a resting position” [95], refuting Taylor et al.'s [12], [17] broad claim. In both the chicken and lizard Varanus exanthematieus radiographs revealed elevation at the base of the neck [95] but that rise is intrinsic to the neck, and evidenced in ONP (Figure 14c). Incidentally, while indeed the chicken neck also rises at the base and is vertical at mid-length, that is achieved without flexion, and some birds even have a horizontal mid-neck in ONP (in fact one that is inverted in the middle, such as the Flightless Cormorant (Phalacrocorax harrisi; Figure 13c).

### Sauropod Necks were Cantilevered

The behavioral claims C0–C4 [12], [17] are not supported by Aves and Crocodylia, let alone Amniota, leaving no EPB support for the conjecture C6. Few vertebrates rest in an alert state with their necks maximally dorsiflexed at the base and heads tucked down maximally, nor are all amniote cervical vertebral columns vertical when they rest (and moreover, many could never achieve such elevation). Lagomorphs and felids are among the relatively few mammals capable of resting with the head “… balanced and supported on top of a straight line which is collinear with the gravity vector” [95]. This ‘sphinx-like’ pose is achieved by dorsiflexing at the cervico-dorsal junction to retract the head, while ventriflexing at the cranio-cervical junction to re-establish a horizontal head (Figure 21a, b). In those vertebrates that can successfully balance the head upon a spring-like vertical column, little further muscular effort is needed to support its weight [95]. Some long necked birds, such as the swan and ostrich, regularly rest with their heads balanced above the base of the neck, which requires significant retraction of the head in the case of the ostrich [15], while others may achieve this in closer to ONP (Figure 13). While some long-necked mammals have sufficiently flexible necks to bend back past vertical, such as giraffes and camels [81], [63]–[64], they do not habitually rest in that pose, since that inverts the head. Maintaining a level head is a behavioral priority [95]–[97] across the Amniota.

While some mammals can and do rest in an alert state by retracting the head to balance it atop a subvertical column, the far more widely-adopted posture in quadrupeds is to cantilever the head and neck before the shoulders, in approximate ONP. The weight of the head and neck is then supported passively by means of suspension through some combination of dorsal musculature and ligaments in tension [114]–[116]. The cervical vertebral column is in low state of flexion (as observed in radiographs of reptiles and birds [95]–[97]). Active dorsiflexion at the base of the neck may further raise the head, of course, depending upon the state of vigilance and alarm (pers. obs.). Again, it is not sufficient to simply cite examples of this behavior in extant vertebrates to support the speculation that sauropods did as well. Osteological correlates, fortunately, have been identified in avian and crocodilian cervical and dorsal morphology [94], [117], [15], [46], [118], [119], [120] which allow an EPB-based inference that at least some sauropods suspended their necks in front of the body. It does not necessarily follow, however, that the neck were held in ONP. Some estimates of head elevation [58], [59] predict higher elevations, and sauropods might have, for purely behavioral reasons, elevated their heads above ONP in the manner in which they are most often illustrated. Head elevation remains ‘intuitively’ logical, recall, as was discussed in the introduction. While it might be tempting to argue based solely examples of extant behavior that support one or the other interpretation, the EPB method builds upon osteological correlates with behavior.

### Evidence regarding the Gravitational Orientation of Sauropod Heads

The osseous labyrinth containing the semicircular canals constitutes a potential osteological correlate for supporting inferences about the preferred or stereotypic head posture in extinct vertebrates [121], [44]. In extant vertebrates the semicircular canals senses angular accelerations in three planes [122], [123]. In the alert state, vertebrates tend to hold the head such that the lateral (or ‘horizontal’) semicircular canal (LSC) is approximately level. If α denotes the angle between the plane of the LSC and the gravitational horizontal, α is usually inclined by roughly 5–10° for many birds and laboratory animals (e.g., domestic cats and rabbits) [124]–[126], [111], [127]–[130], [95]–[97]. The study by de Beer [126] showed a remarkable alignment of the LSC with the horizontal in the alert dog and horse. Larger values of inclination α and more variability in α have been reported for some mammals (rabbit, guinea pig, rat, human) [126], [95]–[97], [124], [131] and some birds such as the spoonbill Platalea and stork Ciconia [111] have negative values of α, i.e., the LSC descends).

The habitual orientation of the head relative to gravity is a behavior property, one that is fortunately correlated with the gravitational orientation of the LSC, and hence of potential use as an osteological correlate [121], [44]. The utility of the LSC for inferring head orientation depends on the quality of the correlation, and by citing the greatest reported range in α (from 30° to −19° [111]), LSC would appear only to give “… only a general idea of the life posture of extinct animals' heads” [12], just as it would make a poor proxy for head orientation in cranial morphometrics [132]. The variation in α across bird taxa reported in Duijm's [111] study (Figure 22a, b) in fact provides a rather more specific idea regarding the orientation of sauropod head orientation.

An EPB-supported inference of the gravitational orientation of the cranium in the extinct vertebrate could be inferred from 1) the observed orientation of the LSC within the cranium of an extinct vertebrate [121], [44], and 2) the inclination angle α in the EPB. The LSC was imaged by μCT for the prosauropod Massospondylus plus the sauropods Diplodocus longus, Camarasaurus lentus and Nigersaurus taqueti [44]. For an assumed α=5°, the four crania could be compared relative to a common frame of reference, namely the LSC (see also [132]). The osteology of the sauropod occiput cranium and atlas-axis is well understood [38], [26], [37], [117], permitting confident estimation of the orientation of the atlas-axis relative to the foramen magnum and the basioccipital condyle. Thus, if the gravitational orientation of the cranium were established, that in turn would indicate the gravitational orientation of the anterior neck. For the prosauropod and the three sauropods studied, the atlas-axis was found to be close to gravitationally horizontal [44] (see Figure 22c, g).

### Combining Independent Lines of Evidence

Proceeding caudally through the occiput (with the basioccipital in articulation with the atlas and the foramen magnum collinear with the neural canal of the atlas-axis), the gravitational slope of the neck at the atlas-axis is constrained as well. Three independent lines of evidence can thus be combined. The LSC data supports a postulated slope for the atlas-axis relative to horizontal, and post-cervical skeletal reconstructions suggest the gravitational slope of the anteriormost dorsal vertebrate (i.e., how the neck emerges from the shoulders), and in the middle, ONP studies of re-articulated cervical columns in the undeflected state, suggest the relative slopes at their two ends. The three lines of evidence combine satisfactorily with the following caveats (all of which are open to eventual EPB-supported verification):

1. Sauropod heads were held in alert rest with a relatively small inclination α of the LSC.
2. Sauropod cranio-cervico joints are held undeflected in alert rest.
3. Sauropod necks are suspended, with intervertebral joints in approximate ONP (i.e., relaxed).
4. Sauropod cervico-dorsal vertebrae are held in approximately ONP in alert rest.

So progressing from the cranium through the cervical column caudally and into the cervico-dorsal transition, a consistent (but still conjectural) global picture is emerging. But speculations about how sauropods held their head in alert rest, when not otherwise occupied, has perhaps less relevance to sauropod biology compared to how the animal used its neck for feeding, and secondarily, while engaged in locomotion.

## Conclusions

Starting with the bare bones, plus caveats about their intervertebral separations based on modern vertebrates with similar articulations, the cervical vertebral columns of sauropods, relieved of their opisthotonic pose (Figure 23), are revealed to be remarkably straight caudally, devoid of any intrinsic sigmoidal-shaped curvature, but some droop cranially (perhaps to re-orient the head ventrally). Osteologically, the base of the neck of all sauropods was a straight collinear extension of the anterior dorsal column. Behaviorally, modern vertebrates, with few exceptions (such as lagomorphs and felids) cantilever the neck and head by dorsal suspension, wherein the intervertebral joints are relaxed and in close to ONP, and the head elevation is that achieved by the ‘pre-formed’ inherent curvature of the cervical column and the slope of the anterior dorsal column at the shoulders.

Sauropod skeletal reconstructions indicate a range of slopes for the anterior dorsal columns. Variation in the resting height and gravitational orientation of the head can be attributed primarily to variations in body plan without postulating any mechanism (either osteological or behavioral) for creating an upward bend in the base of the neck. Thus despite having no intrinsic upward bend at the base of the neck ONP, the sauropod head could have been placed at a substantial elevation above the shoulders, or at or even below the shoulders, simply due to the slope of the anterior dorsal column. Even modest dorsiflexion at the base could then produce several meters of additional head elevation in those sauropods with especially long necks, and those with long necks and high resting height could also ventriflex to bring the head down to browse low (as well as drink water). The once-held distinction between low versus high browsers is not sharply defined.

Upper bounds on neck mobility are predicted geometrically for extant vertebrates, and those criteria, applied to sauropod necks, predict less-than-avian flexibility, presuming sauropod necks did not disarticulate (more than once per lifetime). Extant vertebrates that do not have a sigmoid curve to the neck, sauropod necks were well-suited for directing the head to different locations on a ‘feeding envelope’ surface rather than to any point within the volume within that surface (think cow not swan). While intervertebral flexibility was comparable to most that of reptiles, and less than most birds, they more than made up for ‘stiff’ necks by their absolute length. While some sauropods literally went to extraordinary lengths to sweep out a ‘feeding surface’ swath in front of them by flexing their necks at full extension dorsoventrally and mediolaterally, their necks were neither pre-curved avian-style nor sufficiently flexible to fully explore the volume of space contained within that surface. Despite some uvam acerbam arguments that sauropods held their heads high (based on the alert rest pose for lagomorphs, felids and some ratites) sauropod necks were incapable of the prerequisite ability to retract the head sufficiently to balance it's weight above the shoulders, adopted passive suspension of the head, like extant vertebrates that share this inability. But how sauropods held their head when inactive seems of lesser importance to understanding their feeding behavior (a point underscored by lagomorphs, felids, and some ratites).

Several of the conclusions in this review seem negative, about what sauropod necks did not look like, and what they did not do, and which popularizations are not scientifically supported and should be abandoned. For instance, none were shaped like swan necks, and there is no support for the persistent suggestion they held their heads high habitually. Perhaps the most useful such negative is that sauropods were not unique – at least, there is no evidence to suggest that what is known about the articulation, suspension, and function of extant archosaur vertebral columns does not apply as well to the sauropods, despite their extremes.

All is certainly not negative: there are many EPB-supported (or supportable) hypotheses to propose and to test, given the commonality between extinct and extant archosaurs – inferences which investigators have barely begun to be explore. The lateral semicircular canal evidence is compelling (and likely to become more so), as is the upper-bounds on flexibility implied by vertebrates sensibly preserving a safety factor of overlap at the zygapophyses. Osteological bracing (both its presence and absence) and its relationship to loads imposed upon necks at the limit of flexibility has only been noted in a few cases. The kinematic importance of a sigmoidal intrinsic curve to the neck has been well appreciated for birds, and that extends to extinct vertebrates that share a sigmoidal design. But the implications of a neck that is a simple monotonic arc, devoid of a built-in inflection point, has not been previously explored either in extant vertebrates or in sauropods, and yet clearly has relevance to browse-gathering efficiency and behavior. Preconceived notions and ill-supported presuppositions will be replaced increasingly by newly-conceived notions as methodology replaces mythology in the study of sauropods.

### Some Notes Regarding Digital Modeling

The 3D models that appear in this review have been developed in Autodesk Maya [108] by the author using standard methods of digital modeling and animation. Each model consists of a set of polygonal objects to represent the osteology, and a ‘rig’, i.e., a set of joint nodes [108] to which these objects are parented in a hierarchical fashion, using the industry convention of defining a ‘root joint’ at the sacrum. The axial skeleton then extends cranially and caudally as distinct kinematic chains, along with the left and right hindlimbs, also forming distinct chains, and continuing, and so forth, in accordance with conventional quadrupedal character rigs [108].

**Figure 24:**
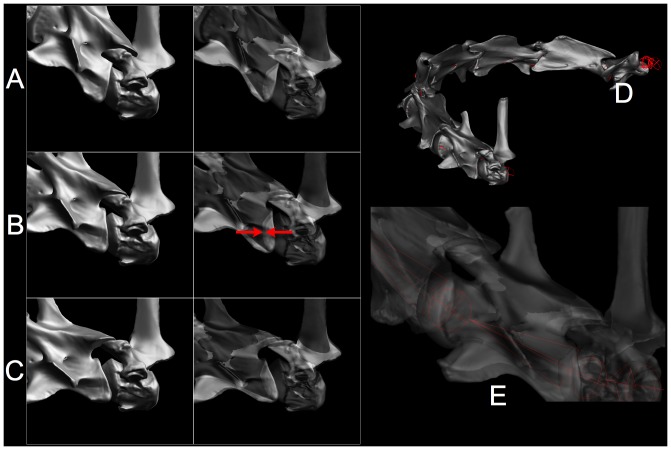
Digital articulation. CT data of individual vertebrae of a recent giraffe Giraffa Camelopardalis are articulated in Autodesk Maya [108]. Cervical vertebra C7 pivots about a center of rotation that closely corresponds to the center of curvature of the roughly hemispherical condyle of T1, confirmed by exploratory manipulation and adjustment, resulting in close intervertebral separations as reported in [15] (see red arrows). In A–C, by alternating between opaque and transparent one can observe osteological bracing dorsiflexion (A) and the ZSF at the limit of ventriflexion. With all intervertebral joints adjusted (D–E), the articulated neck approximates the range of motion observed in life (see also Figures 11, 12). This method applies equally to the similarly opisthocoelous vertebrae [30]–[32], see Figure 25. CT data provided courtesy American Museum of Natural History.

**Figure 25:**
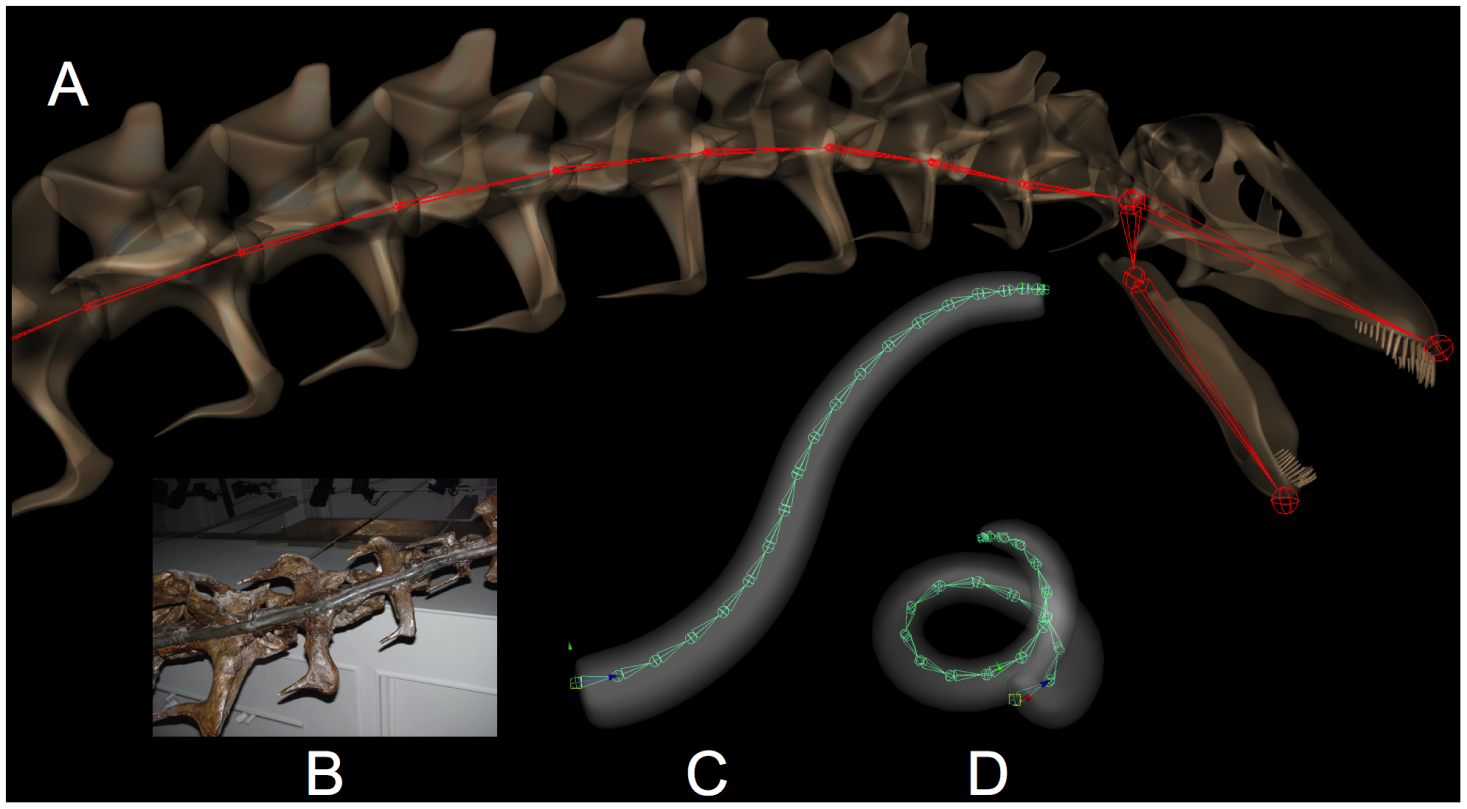
Creating digital, articulated skeletal models. In A, the cervical vertebrae of A. louisae CM 3018, modeled by subdivision surfaces (see text) are rigged to form a kinematic chain with joints at the centers of curvature of the condyles (displayed in red), with empirically-determined intervertebral separations that maximize the congruence between condyles and cotyles and associated zygaphophyseal pairs at each intervertebral joint. The articulated skeleton resembles the original specimen (B), but fortunately without the rigid steel armature. In C, a digital model of an ostrich Struthio camelus is shown in ONP, based on published data [15] of joint-by-joint intervertebral separations and flexion limits (in both mediolateral and dorsoventral flexion), and in D, an example of its extraordinary flexibility. Supplemental material: Movie S11, Movie S12, File S1.

Regarding the modeling of individual bones, conventional digital modeling employes two somewhat disparate choices: importing a polygonal mesh of vertices that form a piecewise planar approximation to a surface from sampled positions across the given object which, given sufficiently many samples, creates an apparently smooth replica of an actual specimens (see the giraffe CT data in Figure 24). The alternative is to create a meshes derived from mathematical representations of smooth surfaces, such as subdivision surfaces [107]. The sauropod models shown here and in Figure 25 are all based on the latter, but individually shaped to closely conform with digitization data when available (but that represents but one resource for creating dimensionally-accurate replicas of the surface morphology of fossil specimens). As in conventional sculpting, a solid form can be approximated from orthographic views (digitally, 2D source images can be superimposed on planes in the 3D modeling space). The primary benefit of using models (rather than ‘real data’ from CT or other digital sources) is permitting the creation of skeletal reconstructions that fill missing elements, provide alternative restorations to damaged specimens, and to correct distortions that are not amenable to automatic retrodeformation techniques [106].

In modeling based on deformable 3D surfaces, a set of prototypical shapes are created, each a generic form (e.g. of a femur, tibia, dorsal vertebra, rib) that represent sufficient morphology to capture the major osteological features (fenestrae, trochanters, laminae, processes, condyles, etc.) sufficient to model a range of variation across taxa for appendicular elements, and across both taxa and position within a vertebral column for axial elements. To model a specific dorsal vertebral column, for example, a generic dorsal vertebra model is duplicated multiple times to represent the first, mid, and last vertebrae of a given specimen. Each instance is then individually sculpted to match the shape and dimensions of its original counterpart, based on archival material, photographs, and, when available, digitized surface scans, CT, or other point-sampled data of actual specimens. Vertebral osteology in archosaurs varies sufficiently smoothly that missing elements can be interpolated over small intervals. Interpolation of missing or severely damaged elements based on adjacent elements is justifiably criticized as being somewhat speculative, as in the case of the restoration of C13–C15 in the Carnegie Museum specimen Apatosaurus louisae CM 3018 [137]. It is conceivable that these vertebrae were not interpolates of their neighboring vertebrae, just as C7 in Giraffe is unique and not predicted as a straightforward interpolate of C6 and T1. But the gradual variation of morphology along sauropod axial skeletons, and the morphological similarity of corresponding cervical vertebrae across known Apatosaurus specimens supports the restoration of missing or damaged elements by interpolation.

Next, blend shape animation [109] permits shape interpolation to form the intermediate vertebrae as blends, to create the complete dorsal series. Due to the gradual progression of morphological variation along a vertebral column in archosaurs, linear shape interpolation between axial elements spaced by four or so vertebrae provides a good first approximation, to be followed by refinement. Finally, after establishing the general trends along the entire axial and appendicular skeletons, specific variations are added based on detailed measurements and comparison with reference material.

The skeleton is then rigged to become fully articulated (following standard rigging practices [108]), yet allowing for subsequent adjustments (e.g., of bone morphology, joints the centers of rotation for all joints including the angulation of ribs to form a ribcage, and pectoral girdle placement). Estimating range of motion in a vertebral column requires estimating the centers of rotation for intervertebral joints (see text) as well as the intervertebral separation. Fortunately, manipulation of digital models in three dimensions permits exploratory confirmation of the center of rotation and spacing between condyle and cotyle essentially simulating realtime fluoroscopy to verify the mechanics of articulation.

Once the rigged skeletal model is complete (but always open to subsequent modification and refinement), the digital joints can be exercised to explore the intervertebral range of motion along the axial skeleton, reachability envelopes, and so forth, as exemplified by the figures in this review.

## Supporting Information

Figure S1 (TIF)

Figure S2 (TIF)

File S1 (ZIP)

Movie S1 (MP4)

Movie S2 (MP4)

Movie S3 (MP4)

Movie S4 (MP4)

Movie S5 (MP4)

Movie S6 (MP4)

Movie S7 (MP4)

Movie S8 A turntable movie depicting the spinal cord (red) entering the foramen magnum of Kaatedocus siberi.
(MP4)

Movie S9 Animation of Camarasaurus from its death pose into a life pose near ONP.
(MP4)

Movie S10 (MP4)

Movie S11 (MP4)

Movie S12 (MP4)

## References

[1] Reisdorf AG, Wuttke M (2012) Re-evaluating Moodie's Opisthotonic- Posture Hypothesis in Fossil Vertebrates Part I: Reptiles – the taphonomy of the bipedal dinosaurs Compsognathus longipes and Juravenator starki from the Solnhofen Archipelago (Jurassic, Germany). Palaeobiodiversity and Palaeoenvironments 92: 119–168 10.1007/s12549-011-0068-y

[2] Tornier G (1909) Wie war der Diplodocus carnegii wirklich gebaut? Sitzungsberichte der Gesellschaft Naturforschender Freunde zu Berlin 1909-4: 193–209.

[3] Hay OP (1910) On the manner of locomotion of the dinosaurs, especially Diplodocus, with remarks on the origin of the birds. Proceedings of the Washington Academy of Sciences. 12: 1–25.

[4] Wiman C (1929) Die Kriede-dinosaurier aus Shantung. Palaeontologica Sinica (Series C) 6: 1–67.

[5] Paul G (1998) Differing bipedal and tripodal feeding modes in sauropods. Journal of Vertebrate Paleontology 69: 70A.

[6] Mazzetta GV, Blanco RE (2001) Speeds of dinosaurs from the Albian-Cenomanian of Patagonia and sauropod stance and gait. Acta Palaeontologica Polonica 46: 235–246.

[7] Myhrvold NP, Currie PJ (1997) Supersonic sauropods? Tail dynamics in the diplodocids. Paleobiology 23: 393–409.

[8] Taylor MP, Wedel MJ, Cifelli RL (2011) A new sauropod dinosaur from the Lower Cretaceous Cedar Mountain Formation, Utah, USA. Acta Palaeontologica Polonica 56: 75–98.

[9] Siegwarth JD, Smith CN, Redman PD (2011) An alternative sauropod physiology and cardiovascular system that eliminates high blood pressures. Lethaia 44: 46–57.

[10] Bakker RT (1978) Dinosaur feeding behavior and the origin of flowering plants. Nature 274: 661–663.

[11] Choy DSJ, Altman P (1992) The cardiovascular system of Barosaurus: and educated guess. Lancet 340: 534–536. 1354287 10.1016/0140-6736(92)91722-k

[12] Taylor MP, Wedel MJ, Naish D (2009) Head and neck posture in sauropod dinosaurs inferred from extant animals. Acta Palaeontologica Polonica 54: 213–220 10.4202/app.2009.0007

[13] Gould SJ (1991) Bully for brontosaurus: reflections in natural history. New York: W.W. Norton. 540.

[14] Paul G (2000) Restoring the life appearance of dinosaurs. In: Paul GS, The Scientific American Book of Dinosaurs. New York: Bryon Press and Scientific American. 78–106.

[15] Dzemski G, Christian A (2007) Flexibility along the neck of the ostrich (Struthio camelus) and consequences for the reconstruction of dinosaurs with extreme neck length. Journal of Morphology 268: 701–714. 17514722 10.1002/jmor.10542

[16] Feynman RP (with Leighton R, Hutchings E) (1985) “Surely you're joking, Mr. Feynman!”: adventures of a curious character. New York: W.W. Norton. 350.

[17] Naish D, Taylor MP, Wedel MJ (2009) Extant animals provide new insights on head and neck posture in sauropods. Journal of Vertebrate Paleontology 29: 56A.

[18] Bryant HN, Russell AP (1992) The role of phylogenetic analysis in the inference of unpreserved attributes of extinct taxa. Philosophical Transactions of the Royal Society of London B 337: 405–418.

[19] Witmer LM (1995) The extant phylogenetic bracket and the importance of reconstructing soft tissues in fossils. In: Thomason JJ, Functional Morphology in Vertebrate Paleontology, Cambridge: Cambridge University Press. 19–33.

[20] Carrano MT, Hutchinson JR (2002) Pelvic and hindlimb musculature of Tyrannosaurus rex (Dinosauria: Theropoda). Journal of Morphology 253: 207–228. 12125061 10.1002/jmor.10018

[21] Moscovici S, Zavalloni M (1969) The group as a polarizer of attitudes. Journal of Personality and Social Psychology 12(2): 125–35.

[22] Chandrashekaran M, Walker BA, Ward JC, Reingen PH (1996) Modeling individual preference evolution and choice in a dynamic group setting. Journal of Marketing Research 33: 211–223.

[23] Myers DG, Lamm H (1976) The group polarization phenomenon. Psychological Bulletin 83: 602–627.

[24] Kassin S, Fein S, Markus HR (2010) Social Psychology. 8th edition. Belmont, CA: Wadsworth Publishing. 613.

[25] Marsh OC (1883) Principal characters of American Jurassic dinosaurs. Pt. VI. Restoration of Brontosaurus. American Journal of Science (series 3) 27: 329–340.

[26] Hatcher JB (1901) Diplodocus (Marsh): its osteology, taxonomy and probable habits, with a restoration of the skeleton. Memoirs of the Carnegie Museum 1: 1–63.

[27] Janensch W (1950) Die Skelettrekonstruktion von Brachiosaurus brancai. Palaeontographica (Suppl. 7)3: 95–103.

[28] Martin J (1987) Mobility and feeding of Cetiosaurus (Saurischia, Sauropoda) why the long neck? In: Currie PJ, Koster EH, Fourth Symposium on Mesozoic Terrestrial Ecosystems, Short Papers. Drumheller: Boxtree Books. 154–159.

[29] McIntosh J, Brett-Surman MK, Farlow JO (1997) Sauropods. In: Farlow JO, Brett-Surman MK, The complete dinosaur. Bloomington: Indiana University Press. 264–290.

[30] Stevens KA, Parrish JM (1999) Neck posture and feeding habits of two Jurassic sauropod dinosaurs. Science 284, April 30: 798–800. 10.1126/science.284.5415.798 10221910

[31] Stevens KA, Parrish JM (2005) Neck posture, dentition and feeding strategies in Jurassic sauropod dinosaurs. In: Tidwell V, Carpenter K, Thunder-lizards: the sauropodomorph dinosaurs. Bloomington: Indiana University Press. 212–232.

[32] Stevens KA, Parrish JM (2005) Digital reconstructions of sauropod dinosaurs and implications for feeding. In: Curry Rogers K, Wilson, J, The Sauropods: Evolution and paleobiology. Berkeley: University of California Press. 178–200.

[33] Christian A (2010) Some sauropods raised their necks: evidence for high browsing in Euhelopus zdanskyi. Biol Lett 6: 823–825 10.1098/rsbl.2010.0359). 20519198 PMC3001369

[34] Clauss M (2011) Sauropod biology and the evolution of gigantism: what do we know? In: Klein N, Remes K, Gee CT, Sander PM, Biology of the Sauropods: understanding the life of gaits. Bloomington: Indiana University Press. 1–7.

[35] Young CC, Zhao X-J (1972) Mamenchisaurus hochuanensis sp. nov. Institute of Vertebrate Paleontology and Paleoanthropology Monographs A 8: 1–30.

[36] Borsuk-Bialynicka M (1977) A new camarasaurid sauropod, Opisthocoelicaudia skarzynskii, n., sp. n., from the Upper Cretaceous of Mongolia. Acta Palaeontologica Polonica 37: 5–64.

[37] Osborn HF, Mook CC (1921) Camarasaurus, Amphicoelias, and other sauropods of Cope. Memoirs of the American Museum of Natural History 3: 247–287.

[38] Gilmore CW (1925) A nearly complete articulated skeleton of Camarasaurus, a saurischian dinosaur from the Dinosaur National Monument. Memoirs of the Carnegie Museum 10: 347–384.

[39] Czerkas SA, Czerkas SJ (1991) Dinosaurs: a global view. New York: Mallard Press. 247.

[40] Christian A, Dzemski G (2007) Reconstruction of the cervical skeleton posture of Brachiosaurus brancai Janensch, 1914 by an analysis of the intervertebral stress along the neck and a comparison with the results of different approaches. Fossil Record 10: 37–48.

[41] Janensch W (1936) Ein aufgestelltes Skelett von Dicraeosaurus hansemanni. Palaeontographica (suppl. 7): 299–308.

[42] Wilson JA (2002) Sauropod dinosaur phylogeny: critique and cladistic analysis. Zoological Journal of the Linnean Society 136: 217–276.

[43] Rauhut OWM, Remes K, Fechner R, Cladera G, Puerta P (2005) Discovery of a short-necked sauropod dinosaur from the Late Jurassic period of Patagonia. Nature 435: 670–672. 15931221 10.1038/nature03623

[44] Sereno PC, Wilson JA, Witmer LM, Whitlock JA, Maga A, et al (2007) Structural extremes in a Cretaceous dinosaur. PLoS ONE 2: e1230 10.1371/journal.pone.0001230) 18030355 PMC2077925

[45] Gilmore CW (1936) The osteology of Apatosaurus with special reference to specimens in the Carnegie Museum. Memoirs of the Carnegie Museum 11: 175–300.

[46] Schwarz D, Frey E, Meyer CA (2007) Novel reconstruction of the orientation of the pectoral girdle in sauropods. The Anatomical Record 290: 32–47. 17441196 10.1002/ar.20405

[47] Taylor MP (2010) Sauropod dinosaur research. Geological Society, London, Special Publications. 343–361–386 doi:10.1144/SP343.22.

[48] Cobley MJ, Rayfield EJ, Barrett PM (2013) Inter-vertebral flexibility of the ostrich neck: implications for estimating sauropod neck flexibility. PLoS ONE 8: e72187 10.1371/journal.pone.0072187 23967284 PMC3743800

[49] Bonnan MF (2003) The evolution of manus shape in sauropod dinosaurs: implications for functional morphology, forelimb orientation, and sauropod phylogeny. Journal of Vertebrate Paleontology 23: 595–613.

[50] Wilhite R (2005) Morphological variation in the appendicular skeleton of North American Upper Jurassic sauropods. In: Tidwell V, Carpenter K, Thunder-lizards: the sauropodomorph dinosaurs. Bloomington: Indiana University Press. 268–301.

[51] Woolnough AP, du Toit JT (2001) Vertical zonation of browse quality in tree canopies exposed to a size-structured guild of Africa browsing ungulates. Oecologia (Berlin) 129: 585–590. 24577699 10.1007/s004420100771

[52] Cameron EZ, du Toit JT (2007) Winning by a neck: tall giraffes avoid competing with shorter browsers. The American Naturalist 169: 130–135. 10.1086/509940 17206591

[53] Seymour RS (2009) Raising the sauropod neck: it costs more to get less. Biology Letters 5: 317–319. 19364714 10.1098/rsbl.2009.0096 PMC2679936

[54] Seymour RS (2009) Sauropods kept their heads down. Science 323: 1671. 19325098 10.1126/science.323.5922.1671

[55] Ganse B, Stahn A, Stoinski S, Suthau T, Gunga H-C (2011) Body mass estimation, thermoregulation, and cardiovascular physiology of large sauropods. In: Klein N, Remes K, Gee CT, Sander PM, Biology of the sauropod dinosaurs: understanding the life of giants. Bloomington: Indiana University Press. 105–115.

[56] Whitlock JA (2011) Inferences of diplodocoid (Sauropoda: Dinosauria) feeding behavior from snout shape and microwear analyses. PLoS ONE 6: e18304 10.1371/journal.pone.0018304 21494685 PMC3071828

[57] Preuschoft H (1976) Funktionelle anpassung evoluierender systeme. Aufsätze und Reden der Senckenbergischen Naturforschender Gesellschaft 28: 98–117.

[58] Christian A, Preuschoft H (1996) Deducing the body posture of extinct large vertebrates from the shape of the vertebral column. Palaeontology 39: 801–812.

[59] Christian A (2002) Neck posture and overall body design in sauropods. Mitteilungen des Museums für Naturkunde Berlin, Geowissenschaftliche Reihe 5: 269–279.

[60] Berman DS, Rothschild BM (2005) Neck posture of sauropods determined using radiological imaging to reveal three-dimensional structure of cervical vertebrae. In: Tidwell V, Carpenter K, Thunder-lizards: the sauropodomorph dinosaurs. Bloomington: Indiana University Press. 233–247.

[61] O'Connor PM (2006) Postcranial pneumaticity: an evaluation of soft-tissue influences on the postcranial skeleton and the reconstruction of pulmonary anatomy in archosaurs. Journal of Morphology. 267: 1199–1226. 16850471 10.1002/jmor.10470

[62] Werner J, Griebeler EM (2013) New insights into non-avian dinosaur reproduction and their evolutionary and ecological implications: linking fossil evidence to allometries of extant close relatives. PLoS ONE 8(8): e72862 10.1371/journal.pone.0072862 23991160 PMC3749170

[63] Dzemski G (2005) Funktionsmorphologische Betrachtung der Halsstellung bei Zoogiraffen. Zool Garten 3: 189–201.

[64] Dzemski G (2006) Funktionsmorphologische Analysen langer Hälse bei rezenten terrestrischen Wirbeltieren zur Rekonstruktion der Stellung und Beweglichkeit langer Hälse prähistorischer Tiere. Ph.D. dissertation. Universität Flensburg Juli.

[65] Van Der Leeuw AHJ, Bout RG, Zweers GA (2001) Evolutionary morphology of the neck system in ratites, fowl, and waterfowl. Netherlands Journal of Zoology 51: 243–262.

[66] Bout RG (1997) Postures of the avian craniocervical column. Journal of Morphology 231: 287–295. 29852617 10.1002/(SICI)1097-4687(199703)231:3<287::AID-JMOR7>3.0.CO;2-8

[67] Bernhardt M, Bridle KH (1989) Segmental analysis of the sagittal plane alignment of the normal thoracic and lumbar spines and thoracolumbar junction. Spine 14: 717–721 10.1097/00007632-198907000-00012 2772721

[68] Sivers W (1934) Ein Beitrag zur Kenntnis des Vogelhalses. Morphologischer Jahrbuecher 74: 697–728.

[69] Boas JEV (1929) Biologisch-anatomische Studien über den Hals der Vogel. Det Kongelige Danske Videnskabernes Selskabs Skrifter 9: 101–222.

[70] Heidweiller J (1989) Postnatal development of the neck system in the chicken (Gallus domesticus). American Journal of Anatomy 186: 258–270. 2618926 10.1002/aja.1001860303

[71] Davies DV, Barnett CH, MacConaill MA (1961) Synovial joints. Their structure and mechanics. Springfield, Illinois: Charles C. Thomas.304.

[72] Kuznetsov AN, Tereschenko VS (2010) A method for estimation of lateral and vertical mobility of platycoelous vertebrae of tetrapods. Paleontological Journal 44: 209–225.

[73] Schmidt H, Heuer F, Claes L, Wilke H-J (2008) The relation between the instantaneous center of rotation and facet joint forces – a finite element analysis. Clinical Biomechanics 23: 270–278. 17997207 10.1016/j.clinbiomech.2007.10.001

[74] Zweers GA, Vanden Berge JC, Koppendraier R (1987) Avian craniocervical systems. I. Anatomy of the cervical-column in the chicken (Gallus gallus L). Acta Morphologica Neerlando- Scandinavica 25: 131–155. 3331053

[75] Pooni JS, Hukins DW, Harris PF, Hilton RC, Davies KE (1986) Comparison of the structure of human intervertebral disks in the cervical, thoracic and lumbar regions of the spine. Surgical and Radiologic Anatomy 8: 175–182. 3099408 10.1007/BF02427846

[76] Bruggeman BJ, Maier JA, Mohiuddin YS, Powers R, Lo Y, Guimarães-Camboa N, Evans SM, Harfe BD (2012) Avian intervertebral disc arises from rostral sclerotome and lacks a nucleus pulposus: implications for evolution of the vertebrate disc. Developmental Dynamics 241: 675–683. 22354863 10.1002/dvdy.23750 PMC3302952

[77] Hall BK (1983) Cartilage, Volume 1. Academic Press. 400.

[78] Malda J, de Grauw JC, Benders KEM, Kik MJ, van der Lest CHA, et al. (2013) Of mice, men and elephants: the relation between articular cartilage thickness and body mass. PLoS ONE. 8(2): e57683. 10.1371/journal.pone.0057683 PMC3578797 23437402

[79] Wedel MJ, Sanders RK (1999) Comparative morphology and functional morphology of the cervical series in Aves and Sauropoda. Journal of Vertebrate Paleontology 19: 83A.

[80] Sereno PC, Beck AL, Moussa B, Dutheil D, Larsson HCE, et al (1999) Cretaceous sauropods from the Sahara and the uneven rate of skeletal evolution among dinosaurs. Science 286: 1342–1347. 10558986 10.1126/science.286.5443.1342

[81] Gauthier-Pilters H, Daag AI (1981) The camel, its ecology, behavior and relationship to man. Chicago: University of Chicago Press. 208.

[82] Klein N, Christian A, Sander PM (2012) Histology shows that elongated neck ribs in sauropod dinosaurs are ossified tendons. Biology Letters 8: 1032–1035. 23034173 10.1098/rsbl.2012.0778 PMC3497149

[83] Schwarz-Wings D, Meyer CA, Frey E, Manz-Steiner HR, Schumacher R (2010) Mechanical implications of pneumatic neck vertebrae in sauropod dinosaurs. Proceedings of the Royal Society of London B 277: 11–17 10.1098/rspb.2009.1275 PMC2842622 19801376

[84] Stevens KA, Wills ED (2001) Gracile versus robust cervical vertebral designs in sauropods. Annual Meeting of the Society of Vertebrate Paleontology, Bozeman, MT. October, 2001. Journal of Vertebrate Paleontology 21: 104A.

[85] Wilson JA (1999) Vertebral laminae in sauropods and other saurischian dinosaurs. Journal of Vertebrate Paleontology 18: 639–653.

[86] Janensch W (1929) Die Wirbelsäule der Gattung Dicraeosaurus hausemanni. Palaeontographica 3 (Suppl. 7)39–133.

[87] Snively E, Cotton JR, Ridgely R, Witmer LM (2013) Multibody dynamics model of head and neck function in Allosaurus (Dinosauria, Theropoda). Palaeontologia Electronica 16 , Issue 2; 11A 29; palaeo-electronica.org/content/2013/389-allosaurus-feeding.

[88] Zhou SH, McCarthy ID, McGregor AH, Coombs RR, Hughes SP (2000) Geometrical dimensions of the lower lumbar vertebrae – analysis of data from digitized CT images. European Spine Journal 9: 242–8. 10905444 10.1007/s005860000140 PMC3611390

[89] Solounias N (1999) The remarkable anatomy of the giraffe's neck. Journal of Zoology 247: 257–268.

[90] Romer AS (1956) Osteology of the Reptiles. Chicago: University of Chicago 772.

[91] Conrad JL (2006) Postcranial skeleton of Shinisaurus crocodilurus (Squamata: Anguimorpha). Journal of Morphology 267: 759–775. 15570597 10.1002/jmor.10291

[92] van Sittert SJ, Skinner JD, Mitchell G (2010) From fetus to adult—an allometric analysis of the giraffe vertebral column. Journal of Experimental Zoology (Molecular and Developmental Evolution) 314B: 469–479. 10.1002/jez.b.21353 20700891

[93] Badlangana NL, Adams JW, Manger PR (2009) The giraffe (*Giraffa camelopardalis*) cervical vertebral column: a heuristic example in understanding evolutionary processes? Zoological Journal of the Linnean Society 155: 736–757.

[94] Frey E (1988) Anatomie des Korperstammes von Alligator mississippiensis Daudin. Staatliches Museum für Naturkunde Serie A, 105.

[95] Vidal PP, Graf W, Berthoz A (1986) The orientation of the cervical vertebral column in unrestrained awake animals. Experimental Brain Research 61: 549–559. 3082659 10.1007/BF00237580

[96] Graf W, de Waele C, Vidal P P (1992) Skeletal geometry in vertebrates and its relation to the vestibular end organs. In: Berthoz A, Graf W, Vidal PP, The Head-neck sensory motor system. Oxford: Oxford University Press. 129–134.

[97] Graf W, de Waele C, Vidal PP (1995) Functional anatomy of the head−neck movement system of quadrupedal and bipedal mammals. Journal of Anatomy 186: 55–74. 7649818 PMC1167272

[98] Muybridge E (1957) Animals in motion. Mineola, NY: Courier Dover Publications. 416.

[99] Leuthold W (1977) African ungulates: a comparative review of their ethology and behavioral ecology. Springer Verlag, Berlin, 307.

[100] Leuthold B, Leuthold W (1972) Food habits of giraffe in Tsavo National Park, Kenya. East African Wildlife Journal 10: 129–141.

[101] Owen-Smith RN (1988) Megaherbivores: the influence of very large body size on ecology. Cambridge University Press, Cambridge. 369.

[102] Pellew R (1984) The feeding ecology of a selective browser, the giraffe (Giraffa camelopardalis tippelskirchi). Journal of Zoology 202: 57–81.

[103] Young T, Isbell L (1991) Sex differences in giraffe feeding ecology: energetic and social constraints. Ethology 87: 79–89.

[104] Stevens KA (2002) DinoMorph: Parametric Modeling of Skeletal Structures. Senckenbergiana Lethaea 82(1): 23–34.

[105] Janensch W (1950) Die Wirbelsäule von Brachiosaurus brancai. Palaeontographica (Suppl. 7)3: 27–93.

[106] Tschopp E, Russo J, Dzemski G (2013) Retrodeformation as a test for the validity of phylogenetic characters: an example from diplodocid sauropod vertebrae. Palaeontologia Electronica 1998: 16.

[107] Catmull E, Clark J (1978) Recursively generated B-Spline surfaces on arbitrary topological meshes. Computer Aided Design 10: 350–355.

[108] Autodesk (2013) Maya - 3D Animation, Visual Effects & Compositing Software [online] http://usa.autodesk.com/maya/ [Accessed 28 August 2013].

[109] Deng Z, Noh J (2008) Computer facial animation: A survey. In: Deng Z, Neumann U, Data-Driven 3D facial animation. London: Springer-Verlag. 1–28.

[110] Zweers GA, Bout RG, Heidweiller J (1994) Motor organization of the avian head-neck system. In: Davies MNO, Green PR, Perception and Motor Control in Birds. Heidelberg: Springer-Verlag. 201–221.

[111] Duijm M (1951) On the head posture in birds and its relation to some anatomical features. II. Proceedings of the Koninklijke Nederlandse Akademie van Wetenschappen, Series C 54: : 202–211, , 260–271.

[112] Pellionisz AJ, Le Goff B, Jaczkó L (1992) Multidimensional geometry intrinsic to head movements around distributed centers of rotation: A neurocomputer paradigm. In: Berthoz A, Graf W, Vidal PP, The Head-Neck Sensory Motor System. New York: Oxford University Press. 158–167.

[113] Preuschoft H, Hohn B, Stoinski S, Witzel U (2011) Why so huge? Biomechanical reasons for the acquisition of large size in sauropod and theropod dinosaurs. In: Klein N, Remes K, Gee CT, Sander PM. Biology of the sauropods: understanding the life of gaits. Bloomington: Indiana University Press. 197–218.

[114] Jouffroy FK (1992) Evolution of the dorsal muscles of the spine in light of their adaptation to gravity effects. In: Berthoz A, Graf W, Vidal PP, The Head-neck sensory motor system. Oxford: Oxford University Press. 22–35.

[115] Slijper EJ (1946) Comparative biologic–anatomical investigations on the vertebral column and spinal musculature of mammals. Verhandelingen der Koninklijke Akademie van Wetenschappen II 42: 1–128.

[116] Alexander RMcN (1985) Mechanics of posture and gait of some large dinosaurs. Zoological Journal of the Linnean Society 83: 1–25 10.1111/j.1096-3642.1985.tb00871.x

[117] Wedel MJ, Cifelli RL, Sanders RK (2000) Osteology, paleobiology, and relationships of the sauropod dinosaur Sauroposeidon. Acta Palaeontologica Polonica 45: 343–388.

[118] Tsuihiji T (2004) The ligament system in the neck of Rhea americana and its implication for the bifurcated neural spines of sauropod dinosaurs. Journal of Vertebrate Paleontology 24: 165–172.

[119] Schwarz D, Frey E, Meyer CA (2007) Pneumaticity and soft-tissue reconstructions in the neck of diplodocid and dicraeosaurid sauropods. Acta Palaeontologica Polonica 52: 167–188.

[120] O'Connor PM (2007) The postcranial axial skeleton of Majungasaurus crenatissimus (Theropoda: Abelisauridae) from the Late Cretaceous of Madagascar. Journal of Vertebrate Paleontology 27: 127–163.

[121] Witmer LM, Chatterjee S, Franzosa J, Rowe T (2003) Neuroanatomy of flying reptiles and implications for flight, posture and behaviour. Nature 425: 950–953. 14586467 10.1038/nature02048

[122] Rabbitt RD, Damiano ER, Grant JW (2004) Biomechanics of the semicircular canals and otolith organs. In: Highstein SM, Fay RR, Popper AN, The vestibular system. New York: Springer Verlag. 153–202.

[123] Cohen B, Raphan T (2004) The physiology of the vestibulo-ocular reflex (VOR). In: Highstein SM, Fay RR, Popper AN, The vestibular system. New York: Springer Verlag. 235–285.

[124] Girard L (1923) Le plan des canaux semi-circulaires horizontaux considéré comme plan horizontal de la tête. Bulletin et Mémoires de la Societe d'Anthropologie de Paris. Series 7(IV): 14–33.

[125] Lebedkin S (1924) Über die Lage des Canalis semicircularis lateralis bei Säugern. Anatomischer Anzeiger 58: 447–460.

[126] de Beer GR (1947) How animals hold their heads. Proceedings of the Linnean Society of London 159: 125–139.

[127] Mazza D, Winterson B (1984) Semicircular canal orientation in the adult resting rabbit. Acta Oto-Laryngologica (Stockholm) 98: 472–480. 10.3109/00016488409107588 6524343

[128] Erichsen JT, Hodos W, Evinger C, Bessette BB, Phillips SJ (1989) Head orientation in pigeons: postural, locomotor and visual determinants. Brain, Behavior and Evolution 33: 268–278. 10.1159/000115935 2758315

[129] Blanks RHI, Curthoys IS, Markham CH (1972) Planar relationships of semicircular canals in the cat. American Journal of Physiology 223: 55–62. 4556936 10.1152/ajplegacy.1972.223.1.55

[130] Spoor F, Zonneveld F (1998) Comparative review of the human bony labyrinth. Yearbook of Physical Anthropology 41: 211–251. 10.1002/(sici)1096-8644(1998)107:27+<211::aid-ajpa8>3.3.co;2-m 9881527

[131] Wilson VJ, Melvill Jones G (1979) Mammalian vestibular physiology. Plenum Press, New York London.

[132] Marugán-Lobón J, Chiappe LM, Farke AA (2013) The variability of inner ear orientation in saurischian dinosaurs: testing the use of semicircular canals as a reference system for comparative anatomy. PeerJ 1: : e124; DOI 10.7717/peerj.124. PMC3740149 23940837

[133] Tschopp E, Mateus O (2012) The skull and neck of a new flagellicaudatan sauropod from the Morrison Formation and its implication for the evolution and ontogeny of diplodocid dinosaurs. Journal of Systematic Palaeontology. doi:10.1080/14772019.2012.746589.

[134] Ostrom JH, McIntosh JS (2000) Marsh's dinosaurs. The collections of Como Bluff. New Haven: Yale University Press. 416.

[135] Madsen, Jr JH, McIntosh JS, Berman DS (1995) Skull and atlas-axis complex of the upper Jurassic sauropod Camarasaurus Cope (Reptilia: Saurischia). Bulletin of Carnegie Museum of Natural History 31: 115.

[136] McIntosh JS, Miles CA, Cloward KC, Parker JR (1996) A new nearly complete skeleton of Camarasaurus. Bulletin of Gunma Museum of Natural History 1: 1–87.

[137] Upchurch P (2000) Neck posture of sauropod dinosaurs. Science 287: 547b.

