# Supplementary figures and images for: The Articulation of Sauropod Necks: Methodology and Mythology
Source: PLoS One. 2013 Oct 30;8(10):e78572. doi: 10.1371/journal.pone.0078572 (PMC3812995; doi:10.1371/journal.pone.0078572)

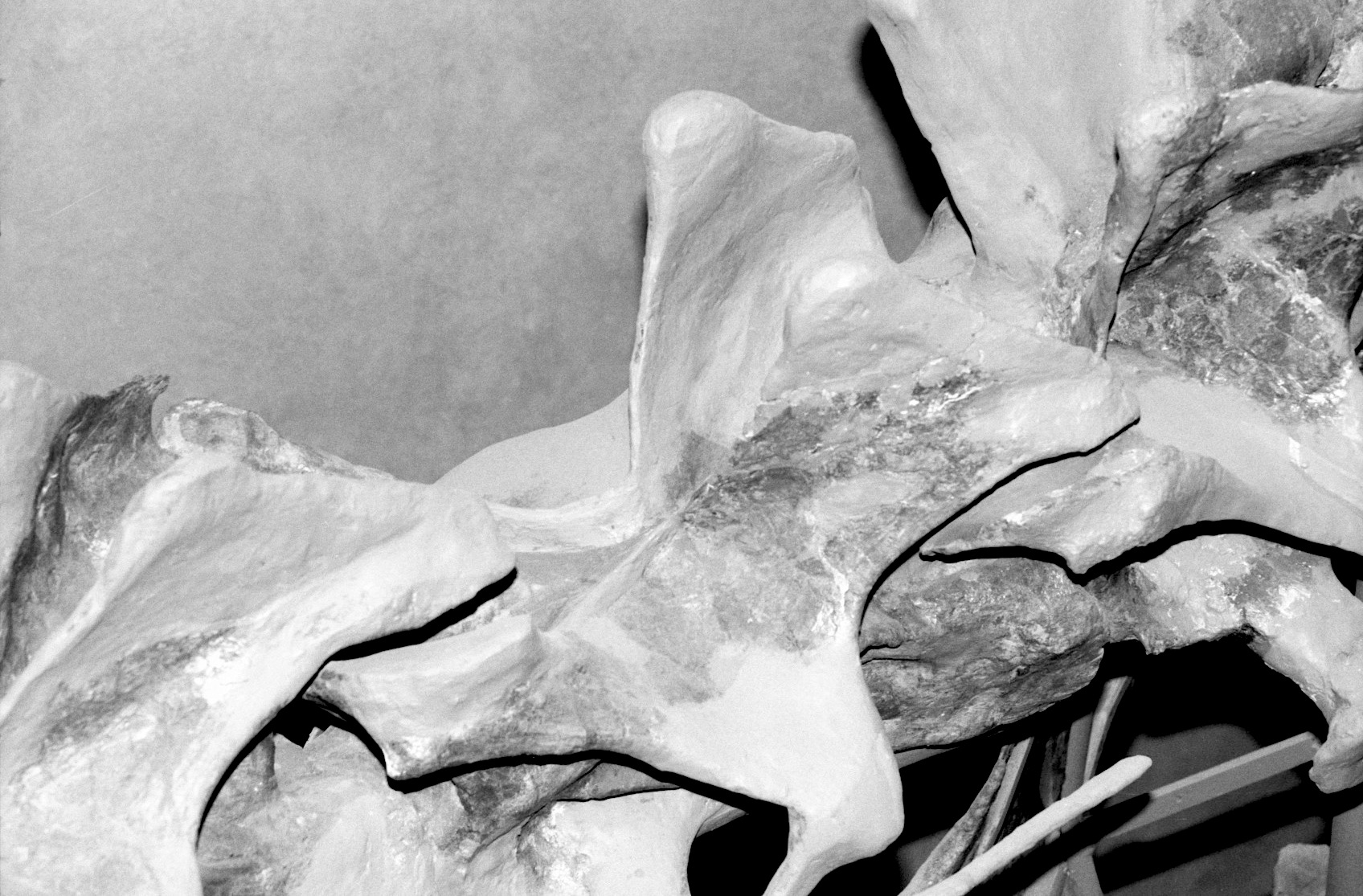

Supplement: Figure S1 — (TIF) [file pone.0078572.s001.tif]

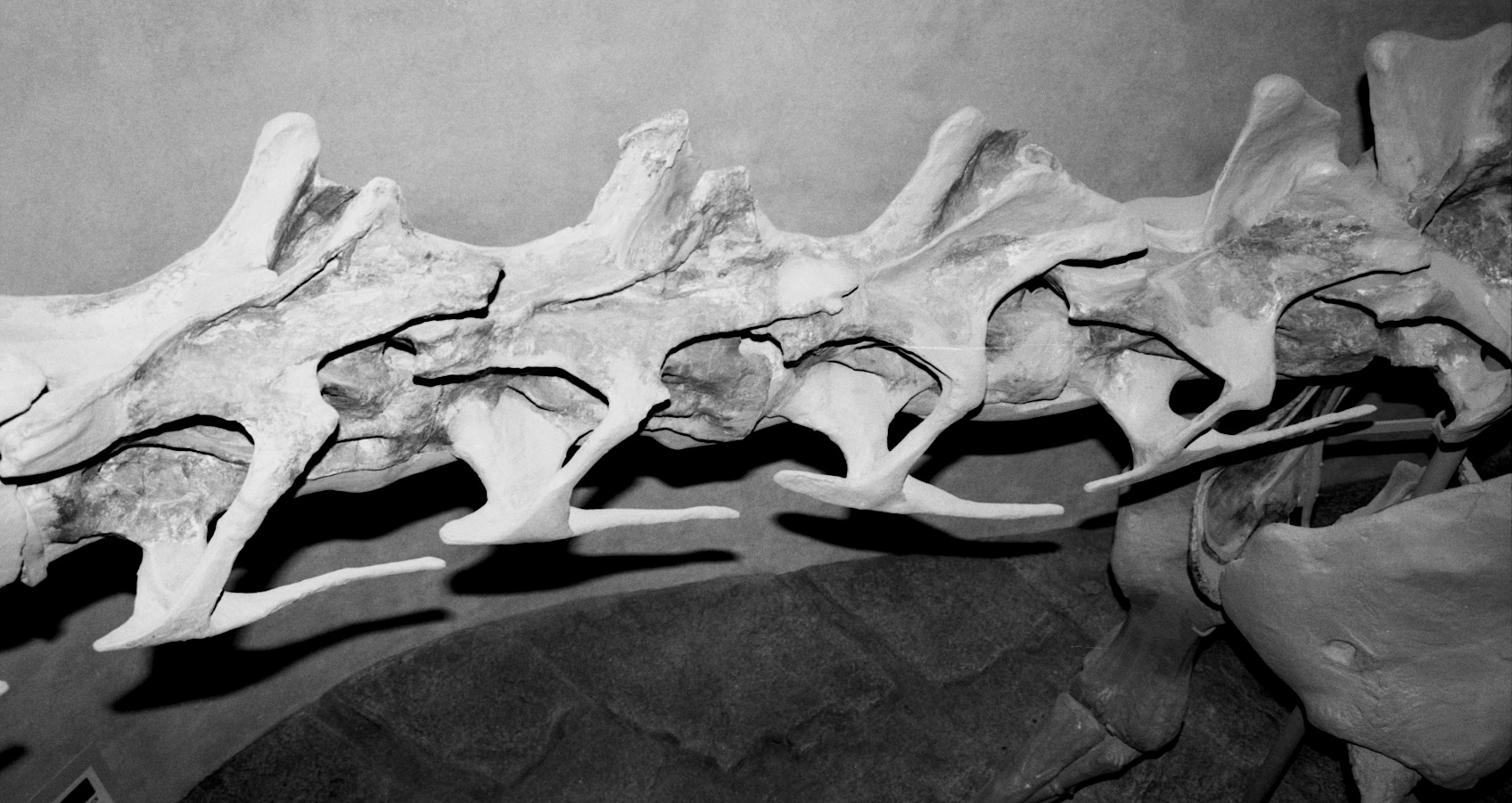

Supplement: Figure S2 — (TIF) [file pone.0078572.s002.tif]
